# Retromer Binding to FAM21 and the WASH Complex Is Perturbed by the Parkinson Disease-Linked VPS35(D620N) Mutation

**DOI:** 10.1016/j.cub.2014.06.024

**Published:** 2014-07-21

**Authors:** Ian J. McGough, Florian Steinberg, Da Jia, Peter A. Barbuti, Kirsty J. McMillan, Kate J. Heesom, Alan L. Whone, Maeve A. Caldwell, Daniel D. Billadeau, Michael K. Rosen, Peter J. Cullen

**Affiliations:** 1 The Henry Wellcome Integrated Signaling Laboratories, School of Biochemistry, Medical Sciences Building, University of Bristol, Bristol BS8 1TD, UK; 2 Department of Biophysics, University of Texas Southwestern Medical Center, Dallas, TX 75390, USA; 3 Henry Wellcome Laboratory for Integrative Neuroscience and Endocrinology, School of Clinical Sciences, University of Bristol, Dorothy Hodgkin Building, Whitson Street, Bristol BS1 3NY, UK; 4 Proteomics Facility, School of Biochemistry, Medical Sciences Building, University of Bristol, Bristol BS8 1TD, UK; 5 Institute of Clinical Neurosciences, University of Bristol, Frenchay Hospital, Bristol BS16 1LE, UK; 6 Departments of Biochemistry and Molecular Biology and Immunology, Mayo Clinic, Rochester, MN 55905, USA; 7 Howard Hughes Medical Institute, University of Texas Southwestern Medical Center, Dallas, TX 75390, USA

## Abstract

Retromer is a protein assembly that plays a central role in orchestrating export of transmembrane-spanning cargo proteins from endosomes into retrieval pathways destined for the Golgi apparatus and the plasma membrane [1]. Recently, a specific mutation in the retromer component VPS35, VPS35(D620N), has linked retromer dysfunction to familial autosomal dominant and sporadic Parkinson disease [2, 3]. However, the effect of this mutation on retromer function remains poorly characterized. Here we established that in cells expressing VPS35(D620N) there is a perturbation in endosome-to-TGN transport but not endosome-to-plasma membrane recycling, which we confirm in patient cells harboring the VPS35(D620N) mutation. Through comparative stable isotope labeling by amino acids in cell culture (SILAC)-based analysis of wild-type VPS35 versus the VPS35(D620N) mutant interactomes, we establish that the major defect of the D620N mutation lies in the association to the actin-nucleating Wiskott-Aldrich syndrome and SCAR homolog (WASH) complex. Moreover, using isothermal calorimetry, we establish that the primary defect of the VPS35(D620N) mutant is a 2.2 ± 0.5-fold decrease in affinity for the WASH complex component FAM21. These data define the primary molecular defect in retromer assembly that arises from the VPS35(D620N) mutation and, by revealing functional effects on retromer-mediated endosome-to-TGN transport, provide new insight into retromer deregulation in Parkinson disease.

## Highlights

• VPS35(D620N) mutation leads to a defect in endosome-to-TGN transport
• Mutant has a perturbed association with the actin-polymerizing WASH complex
• Primary defect is a reduced affinity for binding WASH component FAM21
• Provides new molecular insight into retromer deregulation in Parkinson disease

## Teaser

Using quantitative proteomics and functional analysis in patient-derived fibroblasts, McGough et al. define the primary molecular defect associated with the Parkinson disease-linked retromer VPS35(D620N) mutation and, by revealing effects on retromer-mediated endosome-to-TGN transport, provide new insight into retromer deregulation in this disease.

## Results and Discussion

### Endosome-to-TGN Transport of CI-MPR Is Impaired in VPS35(D620N)-Expressing Cells

**Figure 1:**
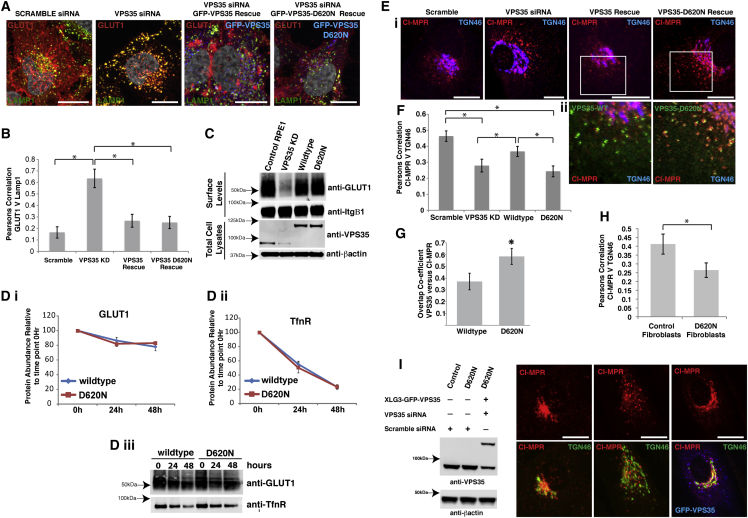
The VPS35(D620N) Mutation Impairs Endosome-to-TGN Transport of CI-MPR (A) GFP-VPS35(D620N) expression rescues the lysosomal accumulation of GLUT1 caused by VPS35 suppression. HeLa cells were transduced with lentiviruses encoding for RNAi-resistant GFP-VPS35 or GFP-VPS35(D620N) and transfected with siRNA-targeting endogenous VPS35 or a nontargeting siRNA. Cells were fixed and stained for VPS35, LAMP1, and GLUT1. Scale bar, 10 μm. (B) The Pearson’s correlation between GLUT1 and Lamp1, in the conditions outlined in (A), quantified from three independent experiments. Approximately 45 cells were analyzed per experiment (^∗^p < 0.01; one-way ANOVA followed by post hoc Tukey test; error bars, SEM). (C) GFP-VPS35(D620N) expression rescues the loss of GLUT1 from the cell surface caused by VPS35 suppression. The surface abundance of GLUT1 in the indicated stable RPE1 cells was determined by quantitative immunoblotting. (Di–Diii) GLUT1 and TfnR degradation kinetics are unaffected by the VPS35(D620N) mutation. The surface of GFP-VPS35 and GFP-VPS35(D620N) cells were randomly biotinylated. Biotinylated proteins were captured from cell lysates with streptavidin beads at indicated time points after biotinylation and subjected to quantitative immunoblotting on an Odyssey scanner. The plots represent the mean of nine independent experiments (error bars, SEM). (Ei, Eii, and F) Steady-state localization of endogenous CI-MPR is perturbed in GFP-VPS35(D620N) RPE1 cells. RPE1 cells suppressed for VPS35 expression and rescued through stable expression of GFP-VPS35 or GFP-VPS35(D620N) were fixed and stained for endogenous CI-MPR (red) and TGN46 (blue). Scale bar, 10 μm. (F) The Pearson’s correlation between CI-MPR and TGN46 from three independent experiments (approximately 45 cells were analyzed per experiment) is shown (^∗^p < 0.01; one-way ANOVA followed by post hoc Tukey test; error bars, SEM). (G) The percentage of overlap between VPS35 or VPS35(D620N) and CI-MPR in the cell periphery (entire cell area excluding the TGN region) is shown in bar graph format. Approximately 20 cells from three independent experiments were analyzed (^∗^p < 0.05; unpaired t test; error bars, SD). (H) Fibroblasts from a healthy donor and a patient harboring the VPS35(D620N) mutation were fixed and stained for endogenous CI-MPR and TGN46. The Pearson’s correlation between CIMPR and TGN46 from three independent experiments (approximately 35 cells were analyzed per experiment) is shown (^∗^p < 0.05; unpaired t test; error bars, SEM). (I) Patient-derived VPS35(D620N) fibroblasts suppressed for endogenous VPS35 and re-expressing wild-type GFP-VPS35 were fixed and stained for endogenous CI-MPR and TGN46. Scale bar, 10 μm.

We sought to investigate the effect of the VPS35(D620N) mutation on trafficking of two known retromer cargos, the endosome-to-TGN transport of the cation-independent mannose 6-phosphate receptor (CI-MPR) [4–7], and the endosome-to-plasma membrane transport of the glucose transporter GLUT1 [8]. At steady state, GLUT1 is localized at the plasma membrane from where it undergoes continuous rounds of endocytosis and PDZ ligand-dependent endosome-to-plasma membrane recycling [9], the latter being mediated by the SNX27-retromer [8]. In the absence of retromer, GLUT1 accumulates in the lysosome and is degraded [8]. To establish whether retromer-mediated endosome-to-plasma membrane transport was affected by the VPS35(D620N) mutation, we performed a quantitative analysis of GLUT1 surface abundance, lysosomal localization, and kinetics of lysosomal-mediated degradation [8]. In HeLa or RPE1 cells, the depletion of endogenous VPS35 by siRNA-mediated suppression followed by re-expression of either wild-type GFP-VPS35 or GFP-VPS35(D620N) produced cell lines where the GFP-tagged VPS35 transgenes were expressed at near to endogenous levels (Figure 1C). In these cells, expression of GFP-VPS35 or GFP-VPS35(D620N) efficiently rescued the lysosomal missorting of GLUT1 observed upon VPS35 suppression (Figures 1A and 1B) (see Figure S1A available online for split channels and Figure S1B for a larger field of view). Furthermore, while the knockdown of VPS35 in RPE1 cells led to a pronounced decrease of GLUT1 surface abundance, re-expression of wild-type or mutant VPS35 rescued GLUT1 surface abundance (Figures 1A and 1C). Finally, an analysis of GLUT1 degradation kinetics in the RPE1 cells revealed that the posttranslational stability of GLUT1 was not affected by the VPS35(D620N) mutation (Figures 1Di–1Diii): the degradation of transferrin receptor was also monitored as a negative control and as expected its degradation rate was also unaffected by the VPS35(D620N) mutation. Overall, these data establish that the VPS35(D620N) mutation does not impair retromer-mediated endosome-to-plasma membrane transport of GLUT1.

Next, we examined the endosome-to-TGN transport of the CI-MPR. After delivery to endosomes, CI-MPR dissociates from its ligand and is recognized by the retromer complex and retrieved to the TGN for further rounds of ligand binding and transport [4–7]. In the absence of retromer, the efficiency of CI-MPR retrieval is perturbed and an increase in endosomal localization of CI-MPR is observed [4–7]. At steady state in our RPE1 cell line, CI-MPR was predominantly localized to the TGN (Figure 1Ei). Suppression of VPS35 led to an increase in the amount of CI-MPR on dispersed puncta and a decrease in the Pearson’s correlation between CI-MPR and TGN46 (Figures 1Ei and 1F). This dispersal and decrease in Pearson’s correlation was partially rescued by re-expressing GFP-VPS35 but not GFP-VPS35(D620N) (Figures 1Ei and 1F). The punctate CI-MPR staining in the VPS35(D620N)-expressing cells was positive for VPS35 (Figure 1Eii), and there was an increase in the overlap between CI-MPR and VPS35 in the GFP-VPS35(D620N) cells when compared to GFP-VPS35-expressing cells (Figure 1G), consistent with a defect in endosome-to-TGN transport and a corresponding CI-MPR dispersal. To extend this, we also examined the steady-state distribution of CI-MPR in fibroblasts obtained from a healthy donor and from a patient harboring the VPS35(D620N) mutation. Again an increased dispersal of CI-MPR was observed (Figures 1H and 1I). This dispersal was not due to a fragmented Golgi as both TGN46 and GRASP65 distribution appeared normal in VPS35(D620N) fibroblasts (Figure S1C). The CI-MPR dispersal observed in the VPS35(D620N) fibroblasts could be rescued by suppression of endogenous VPS35 and re-expression of wild-type GFP-VPS35 (Figure 1I), suggesting that the differences in CI-MPR distribution are not due to variability between cell lines isolated from different patients. These data confirm and extend recent findings of an impairment in retromer-mediated endosome-to-TGN transport in cells carrying the VPS35(D620N) mutation [10, 11].

### The VPS35(D620N) Mutant Does Not Perturb the Formation of the Retromer Heterotrimer or the Endosome Localization of VPS35

**Figure 2:**
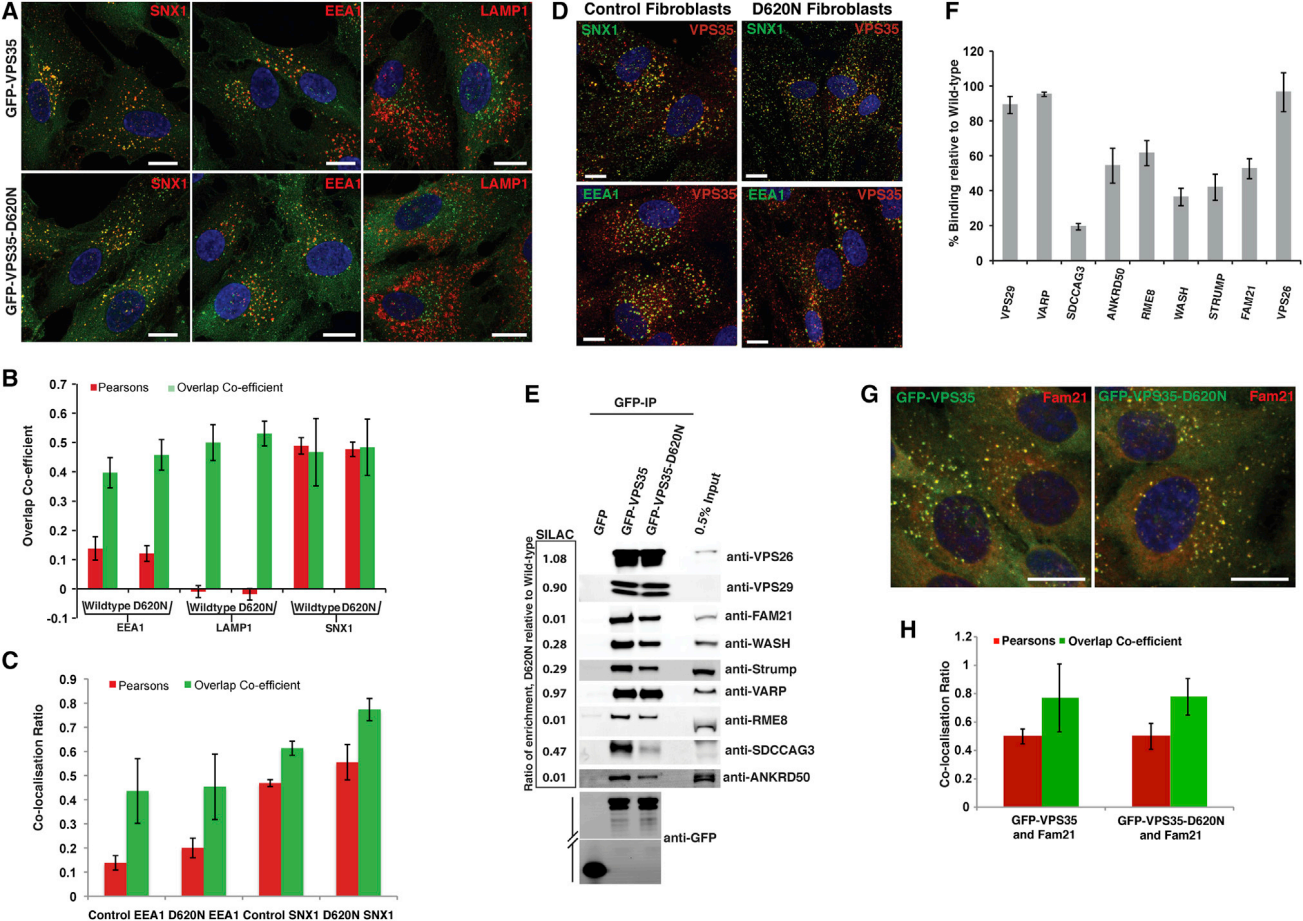
The D620N Mutation in VPS35 Results in a Decrease in Association with the WASH Complex (A and B) Endosomal localization of VPS35 is not perturbed by the VPS35(D620N) substitution. GFP-VPS35- and GFP-VPS35(D620N)-expressing cells were fixed and stained for SNX1, EEA1, and LAMP1 (A). The percentage of overlap and Pearson’s correlation are shown in bar graph format (15 fields of view [approximately 150 cells] from three independent experiments were analyzed; error bars, SEM) (B). Scale bar, 10 μm. (C and D) Fibroblasts from a healthy donor and a patient harboring the VPS35(D620N) mutation were fixed and stained for endogenous SNX1, EEA1, and VPS35 (D). The percentage overlap and Pearson’s correlation are shown in bar graph format (approximately 40 cells from three independent experiments were analyzed; error bars, SEM) (C). Scale bar 10 μm. (E) GFP-VPS35(D620N) immunoprecipitates WASH complex components to a lesser extent than wild-type VPS35. SILAC-based comparative proteomic analysis of GFP-trap-precipitated GFP versus GFP-VPS35 and GFP-VPS35(D620N). The GFP-VPS35, GFP-VPS35(D620N), and GFP stable RPE1 cell lines were grown in normal (GFP), medium-isotope-labeled (VPS35) medium, and heavy-isotope-labeled (VPS35(D620N)) medium, followed by lysis and precipitation with GFP-trap beads. Precipitates were pooled, separated by SDS-PAGE, and subjected to gel walking LC–MS/MS analysis on an Orbitrap mass spectrometer. The SILAC ratio is the fold-enrichment of proteins in GFP-VPS35(D620N) precipitates over GFP-VPS35. Quantitative fluorescent-based immunoblot analysis of precipitates from the stable RPE1 cell lines broadly confirmed and correlated with the SILAC data. (F) The immunoprecipitates were repeated three times and the mean fluorescent intensity of selected interactors in the GFP-VPS35(D620N) immunoprecipitate compared to the corresponding intensity in the GFP-VPS35 immunoprecipitate are shown (error bars, SEM). (G and H) Endosomal localization of FAM21 is not perturbed by the VPS35(D620N) substitution. GFP-VPS35- and GFP-VPS35(D620N)-expressing cells were fixed and stained for FAM21 (G). The Pearson’s correlation and overlap coefficient are shown in bar graph format (40 cells) from three independent experiments were analyzed (error bars, SEM) (H).

To determine the molecular basis of the perturbed endosome-to-TGN trafficking observed with the VPS35(D620N) mutant, we first sought to determine whether the VPS35(D620N) mutation affected formation of the VPS26-VPS29-VPS35 heterotrimer or its endosomal localization. Like GFP-VPS35, GFP-VPS35(D620N) localized predominantly to a sorting nexin-1 (SNX1) positive early-to-late transitional endosomal compartment (Figures 2A and 2B). In agreement with this, VPS35 showed normal distribution and appearance of the retromer-decorated endosomal compartment in the fibroblast cells derived from patient samples as well as comparable colocalization with SNX1 and the early endocytic marker EEA1 (Figures 2C and 2D). Immunoisolation followed by western blotting also established that VPS35(D620N) retained the ability to form a heterotrimeric complex with VPS29 and VPS26A (Figure 2E). These data establish that the molecular defect associated with the VPS35(D620N) mutation lies outside of heterotrimeric retromer assembly and retromer’s endosome association.

### Comparative Interactome Analysis of VPS35 and the VPS35(D620N) Mutant

Retromer scaffolds the assembly of a number of accessory proteins that aid its role in endosomal sorting [1]. To identify the effect of the VPS35(D620N) mutant on the assembly of these accessory proteins, we performed an unbiased quantitative analysis of the VPS35 and VPS35(D620N) interactomes. In a triple SILAC setup, RPE1 cell lines stably expressing GFP, GFP-VPS35 or GFP-VPS35(D620N) were grown in light (GFP), medium heavy (GFP-VPS35) and heavy (GFP-VPS35(D620N)) SILAC media until full steady-state labeling was ensured. Cells were then subjected to lysis, GFP trap immunoisolation and pooling of the samples for SDS-PAGE, and subsequent quantification by LC-MS/MS after in-gel tryptic digestion. Known VPS35-interacting partners VPS26A and VPS26B, VPS29, TBC1D5 [12], SNX27 [8], and all components of the actin-polymerizing Wiskott-Aldrich syndrome and SCAR homolog (WASH) complex [12–17] were enriched in the wild-type GFP-VPS35 immunoisolation (Tables S1 and S2). In addition, a number of previously unidentified interactors including VARP, SDCCAG3, and ANKRD50 were identified, the functional relevance of which will be described elsewhere.

While there is comparative enrichment of VPS29, VPS26A, and VPS26B between wild-type and VPS35(D620N) (left side of Figure 2E; Tables S1 and S2), there was a clear decrease in the enrichment of all components of the WASH complex within the VPS35(D620N) interactome (Figure 2E; Tables S1 and S2). In addition, there was a decrease in enrichment of the known retromer interactor RME-8 [18, 19] and two of the newly identified interactors SDCCAG3 and ANKRD50 (Figure 2E; Tables S1 and S2). In contrast, there was no loss in binding to other known retromer interactors such as TBC1D5 [20] and SNX27 [8] and the newly identified interactor VARP [12] (Figure 2E; Tables S1 and S2). Validating the mass spectrometric quantification, fluorescence-based quantitative western analysis confirmed the decrease in binding of these proteins to the VPS35(D620N) mutant (Figures 2E and 2F). Given the loss of binding to the WASH complex observed in the GFP-VPS35(D620N) cells, we sought to investigate whether the membrane association of the WASH complex is impaired in the VPS35(D620N)-expressing cells. We saw no obvious impairment of FAM21 endosomal localization or a decrease in Pearson’s correlation between GFP-VPS35(D620N) and FAM21 (Figures 2G and 2H). However, we cannot rule out that the loss of binding to the WASH complex alters turnover of the WASH complex on the endosome or affects the endosomal subdomain organization of the retromer and WASH complexes.

### The Binding of ANKRD50, SDCCAG3, and RME-8 to Retromer Is Mediated through the WASH Complex

**Figure 3:**
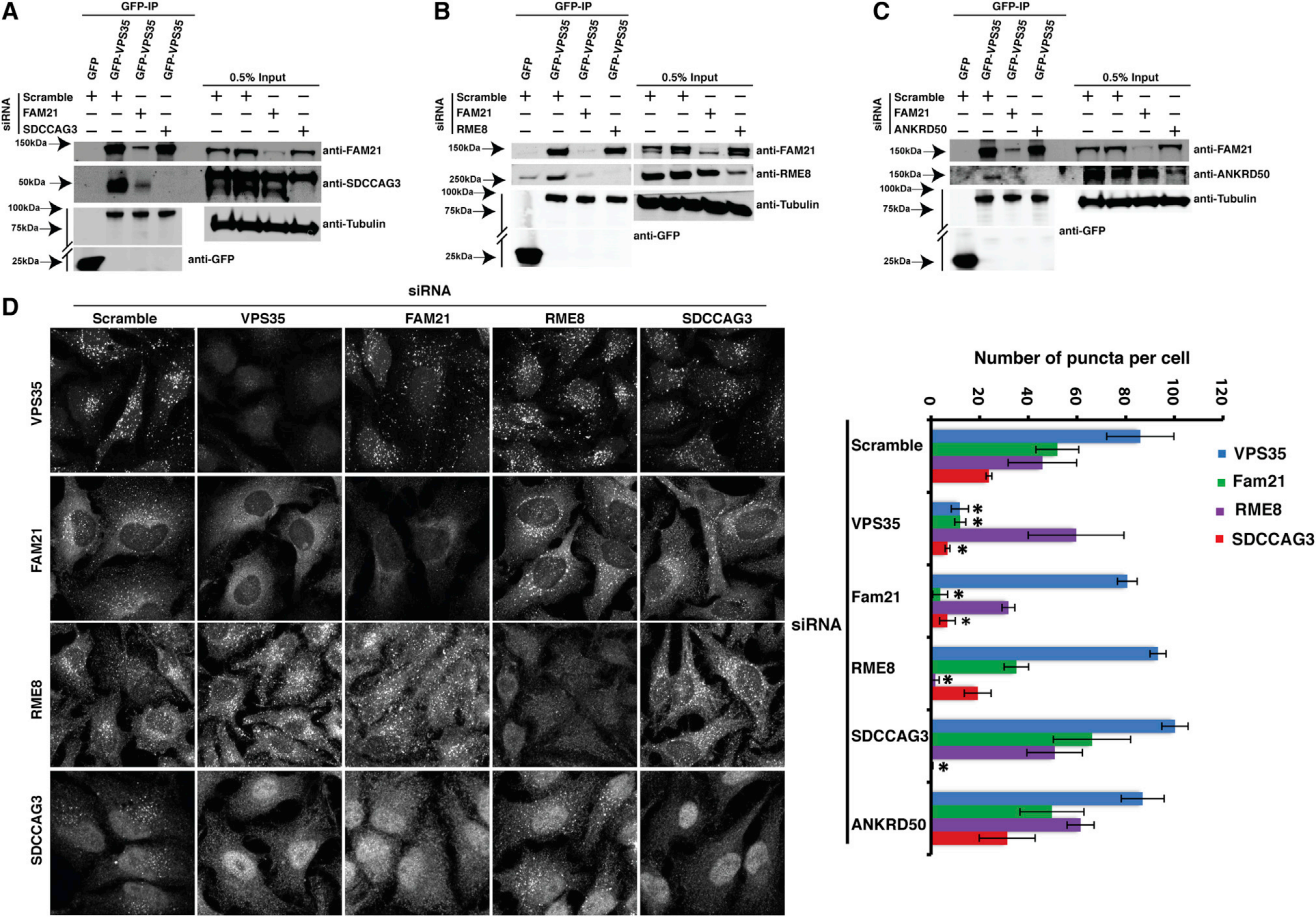
The WASH Complex Is Required for the Association of RME8, SDCCAG3, and ANKRD50 with VPS35 (A, B, and C) GFP-VPS35-expressing RPE1 cells were transfected with nontargeting siRNA or siRNA against FAM21, RME-8, SDCCAG3, or ANKRD50 prior to GFP-trap immunoprecipitation of GFP-VPS35 and immunoblotting with antibodies raised against FAM21, SDCCAG3, RME-8, ANKRD50, Tubulin, and GFP. Suppression of FAM21 resulted in a loss of SDCCAG3 (A), RME-8 (B), and ANKRD50 (C) from VPS35 immunoprecipitates. Suppression of RME-8, SDCCAG3, or ANKRD50 did not affect the ability of VPS35 to immunoprecipitate FAM21. (D) FAM21 and retromer are required for the endosomal localization of SDCCAG3. HeLa cells were transfected with nontargeting siRNA or siRNA against VPS35, FAM21, RME-8, or SDCCAG3 prior to imaging of the endogenous localization of each protein. Scale bar, 10 μm. (E) The number of puncta per cell above a set threshold intensity, from three independent experiments (30 cells per experiment), for the indicated proteins was quantified using Volocity (^∗^p < 0.01; one-way ANOVA followed by post hoc Tukey test; error bars, SD).

To identify the primary defect in the VPS35(D620N) mutant, we performed a series of GFP-VPS35 immunoisolations from cells treated with RNAi’s targeting FAM21—the component of the WASH complex that directly binds to VPS35 [16], ANKRD50, SDCCAG3, and RME-8. Suppression of FAM21 resulted in a loss in the enrichment of ANKRD50, SDCCAG3, and RME-8 in GFP-VPS35 immunoisolates (Figures 3A–3C), establishing that these proteins interact indirectly with retromer through the WASH complex. Suppression of ANKRD50, SDCCAG3, and RME-8 did not affect the binding of FAM21 to VPS35 (Figures 3A–3C).

To correlate these data with the previously described endosome association of SDCCAG3 and RME-8 [21, 22], we analyzed parallel experiments using immunofluorescence. Confirming published data, suppression of VPS35 led to the dissociation of FAM21 from endosomes (Figures 3D and 3E) [15, 16]. Consistent with the biochemical analysis, the endosome association of SDCCAG3 required the presence of both retromer and FAM21 (Figures 3D and 3E). In contrast, the endosome association of RME-8 was only partially dependent on retromer and FAM21 (Figures 3D and 3E), which is consistent with the known binding of RME-8 to other endosome-associated proteins including SNX1 [18]. Together, these data establish that the association of ANKRD50, SDCCAG3, and RME-8 to retromer is mediated through binding to the WASH complex, and for SDCCAG3 this is necessary for targeting to retromer-labeled endosomes. Moreover, they provide evidence that the major defect of the VPS35(D620N) mutant lies with the association to the WASH complex.

The VPS35(D620N) mutation has been suggested to inhibit the neuroprotective effect of VPS35 [23], so we sought to investigate whether suppression of VPS35, FAM21, SDCCAG3, RME-8, or ANKRD50 enhanced the toxicity and increased cell death observed in the neuroblastoma cell line SH-SY5Y upon exposure to MPP+, which interferes with complex I of the electron transport chain in mitochondrion, causing cell death. However, no increase in cell death upon suppression of any of these components was observed (Figure S2) when the cells were incubated in 500 μM of MPP+ for 48 hr. This is in line with a previous study that found no effect of VPS35 suppression on cell survival when SH-SY5Y cells were challenged with MPP+ [23]. However, the absence of an effect could also be due to insufficient suppression of these components in SH-SY5Y cells.

### VPS35(D620N) Impairs Its Interaction with FAM21 In Vitro

Previous studies have suggested that the interaction between the retromer and FAM21 is mediated by the VPS35 and VPS29 (but not VPS26) subunits of the retromer heterotrimer and multiple LFa motifs from the C terminus of FAM21 [15, 16, 24]. FAM21 contains 21 such LFa motifs with varied affinities to the VPS35/VPS29 complex. The two most C-terminal ones, R20 and R21, bind to the VPS35/VPS29 complex with the highest affinity, and deletion of R20-21 abolished endosomal localization of FAM21 in cells [16]. Thus, to evaluate the effect of the VPS35(D620N) substitution, we examined whether this mutation affects the affinity of the VPS35/VPS29 complex to the R20-21 fragment by using isothermal titration calorimetry (ITC).

**Figure 4:**
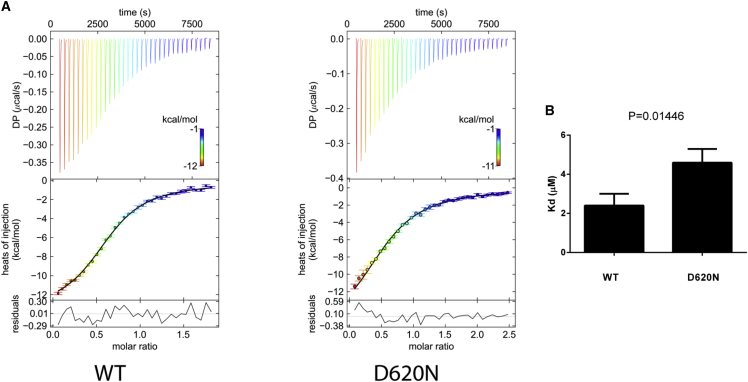
VPS35(D620N) Has Reduced Affinity for the FAM21 Tail (A) Isothermal titration calorimetry of FAM21_R20-21_ titrated into the VPS35/VPS29 complex. Top, middle, and bottom panels are from representative experiments showing raw, integrated heat, and fitting residuals, respectively. The black curve in the middle panel represents a fit of the integrated data. Left: VPS35^WT^/VPS29. Right: VPS35^D620N^/VPS29. (B) Quantification of the dissociation constant (K_d_) determined from three independent ITC experiments for each protein complex as shown in (A). All values are presented as mean ± SD, derived from software package SEDPHAT for a global fitting. Student’s t test was used to determine the difference of the two sets of data.

The recombinant VPS35/VPS29 complex was purified by colysing individually expressing bacterial cells as described previously [16]. The VPS35(D620N) substitution did not affect complex formation with VPS29, further confirming our biochemical and proteomic analysis (Figures 2E and 2F). The ITC experiments for each VPS35/VPS29 complex were repeated three times with two independently prepared protein samples and slightly different injection protocols. Raw data were pooled and subjected to global fitting using the software package SEDPHAT. Similar to our previous studies, VPS35/VPS29 bound R20-21 with a stoichiometry of 1:2 [16]. The affinity of VPS35(D620N)/VPS29 to R20-21 (K_d_ = 4.6 ± 0.7 μM) was significantly lower (by 2.2 ± 0.5-fold) than that of the wild-type VPS35/VPS29 (K_d_ = 2.4 ± 0.6 μM) (Figures 4A and 4B). These results establish that the VPS35(D620N) substitution impairs its interaction with FAM21.

In addition to R20-21, we also attempted to measure the affinity for a larger fragment of FAM21, R15-21. Although the ITC thermograms were qualitatively consistent with a reduced affinity for the VPS35(D620N) mutant, since the R15-21 fragment contains seven LFa motifs potentially interacting with VPS35/VPS29, it was not possible to quantitatively fit and interpret these data (data not shown).

In summary, stemming from an unbiased quantitative proteomic analysis, we have identified that at the molecular level the primary effect of the Parkinson disease-linked VPS35(D620N) mutation is a decrease in the affinity for FAM21 and as a consequence a reduced association with the WASH complex and its associated proteins, including RME-8, and the newly identified retromer-associated proteins SDCCAG3 and ANKRD50. While not perturbing retromer-mediated endosome-to-plasma membrane recycling of GLUT1, this partial loss of FAM21 binding and the WASH complex-associated proteins does lead to a decrease in the efficiency of endosomes-to-TGN retrieval of the CI-MPR. While the primary defect of the VPS35(D620N) mutation is a decrease in affinity for the WASH complex, it is important to stress that the observed phenotypes maybe arise from a reduced activity of RME8, SDCCAG3, or ANKRD50 or a combination of these and FAM21. This is consistent with the current view that WASH-mediated actin polymerization is required for the organization and maturation of retromer sorting domains on early-to-late transition endosomes [16]. Precisely why the decrease in coupling to the WASH complex, and associated proteins, leads to a selective retromer effect on endosome-to-TGN over endosome-to-plasma membrane transport is unclear and will require further investigation. With evidence linking mutations in the WASH complex component strumpellin to hereditary spastic paraplegia [25], and the recent description of an RME-8(N855S) mutation that segregates with late-onset Parkinson disease [26], the data presented here establish that the association and function relationship between retromer and WASH complexes will be an important area in defining the role of defective endosomal sorting in these neurodegenerative diseases.

During the final stages of reviewing our manuscript Zavodszky and colleagues, using a candidate approach, published work consistent with a defect in WASH binding to the VPS35(D620N) mutant [27]. Our detailed biochemical analysis extends their findings and, importantly, through our unbiased global analysis, highlights that the corresponding loss of RME-8, SDCCAG3, and/or ANKRD50 may also contribute to the defect in retromer function in Parkinson disease. As such, the role of these newly identified retromer interactors offers a potentially exciting avenue of research into the causes of this neurodegenerative disease.

## Author Contributions

I.J.M, F.S., D.D.B., M.K.R., and P.J.C. designed the project; I.J.M. and F.S. performed the cell biology experiments; K.J.H. performed the proteomics; D.J. performed the biochemical analysis of the VPS35 FAM21 interaction; P.A.B., K.J.M., A.L.W., and M.A.C. generated the VPS35(D620N) patient fibroblast cell line. I.J.M., F.S., D.J., D.D.B., M.K.R., and P.J.C. wrote the manuscript.

## Supplemental Information

Document S1. Supplemental Experimental Procedures and Figures S1 and S2

Table S1. SILAC Quantified Interactome GFP-VPS35 versus GFPVPS35(D620N) from Human RPE1 Cells Filtered to Include Only Top Heights, the Cutoff for Filtering Was an Enrichment of 5-fold or More and with Two or More Peptides Quantified Human RPE1 cells expressing either GFP, GFP-VPS35, or GFPVPS35(D620N) were cultured in light DMEM (GFP), medium heavy (GFP-VPS35), or heavy SILAC medium (GFP-VPS35(D620N)), followed by lysis and precipitation with GFP Nanotrap beads. After precipitation, beads were combined, bound proteins resolved by SDS-PAGE, and subjected to detection and quantification of heavy and light peptides by LC-MS/MS.

Table S2. Unfiltered SILAC Quantified Interactome GFP-VPS35 versus GFP-VPS35(D620N) from Human RPE1 Cells The same data set as in Table S1 but without the cutoff for enrichment and number of peptides detected. Human RPE1 cells expressing either GFP, GFP-VPS35, or GFPVPS35(D620N) were cultured in light DMEM (GFP), medium heavy (GFP-VPS35), or heavy SILAC medium (GFP-VPS35(D620N)), followed by lysis and precipitation with GFP Nanotrap beads. After precipitation, beads were combined, bound proteins resolved by SDS-PAGE, and subjected to detection and quantification of heavy and light peptides by LC-MS/MS.

Document S2. Article plus Supplemental Information
