# Retromer Binding to FAM21 and the WASH Complex Is Perturbed by the Parkinson Disease-Linked VPS35(D620N) Mutation

**DOI:** 10.1016/j.cub.2014.07.004

**Published:** 2014-07-21

**Authors:** Ian J. McGough, Florian Steinberg, Da Jia, Peter A. Barbuti, Kirsty J. McMillan, Kate J. Heesom, Alan L. Whone, Maeve A. Caldwell, Daniel D. Billadeau, Michael K. Rosen, Peter J. Cullen

**Affiliations:** 1 The Henry Wellcome Integrated Signaling Laboratories, School of Biochemistry, Medical Sciences Building, University of Bristol, Bristol BS8 1TD, UK; 2 Department of Biophysics, University of Texas Southwestern Medical Center, Dallas, TX 75390, USA; 3 Henry Wellcome Laboratory for Integrative Neuroscience and Endocrinology, School of Clinical Sciences, University of Bristol, Dorothy Hodgkin Building, Whitson Street, Bristol BS1 3NY, UK; 4 Proteomics Facility, School of Biochemistry, Medical Sciences Building, University of Bristol, Bristol BS8 1TD, UK; 5 Institute of Clinical Neurosciences, University of Bristol, Frenchay Hospital, Bristol BS16 1LE, UK; 6 Departments of Biochemistry and Molecular Biology and Immunology, Mayo Clinic, Rochester, MN 55905, USA; 7 Howard Hughes Medical Institute, University of Texas Southwestern Medical Center, Dallas, TX 75390, USA

(Current Biology *24*, 1670–1676; July 21, 2014)

In the version of this article originally published online, Dr. Alan L. Whone was omitted from the author list. Dr. Whone and his affiliation have now been added to the article online and in print; the correct author list is shown above as well. The Author Contributions section of the article has also been updated to reflect Dr. Whone’s contributions. The authors sincerely apologize to Dr. Whone for the omission.

Additionally, we would like to take this opportunity to include mention of a study that reported the interaction between RME-8 and FAM21; this work was published after the submission of our original manuscript:

Freeman, C.L., Hesketh, G., and Seaman, M.N. (2014). RME-8 coordinates the activity of the WASH complex with the function of the retromer SNX dimer to control endosomal tubulation. J. Cell Sci. *127*, 2053–2070.

