# Supplementary material for: Retromer Binding to FAM21 and the WASH Complex Is Perturbed by the Parkinson Disease-Linked VPS35(D620N) Mutation
Source: Curr Biol. 2014 Jul 21;24(14):1670–6. doi: 10.1016/j.cub.2014.06.024 (PMC4110399; doi:10.1016/j.cub.2014.06.024)
Supplement: Document S1. Supplemental Experimental Procedures and Figures S1 and S2 [file mmc1.pdf]

Current Biology, Volume *24*

Supplemental Information

Retromer Binding to FAM21 and the WASH Complex Is Perturbed by the Parkinson Disease-Linked VPS35(D620N) Mutation

Ian J. McGough, Florian Steinberg, Da Jia, Peter A. Barbuti, Kirsty J. McMillan, Kate J. Heesom, Alan L. Whone, Maeve A. Caldwell, Daniel D. Billadeau, Michael K. Rosen, and Peter J. Cullen

Supplementary Figures

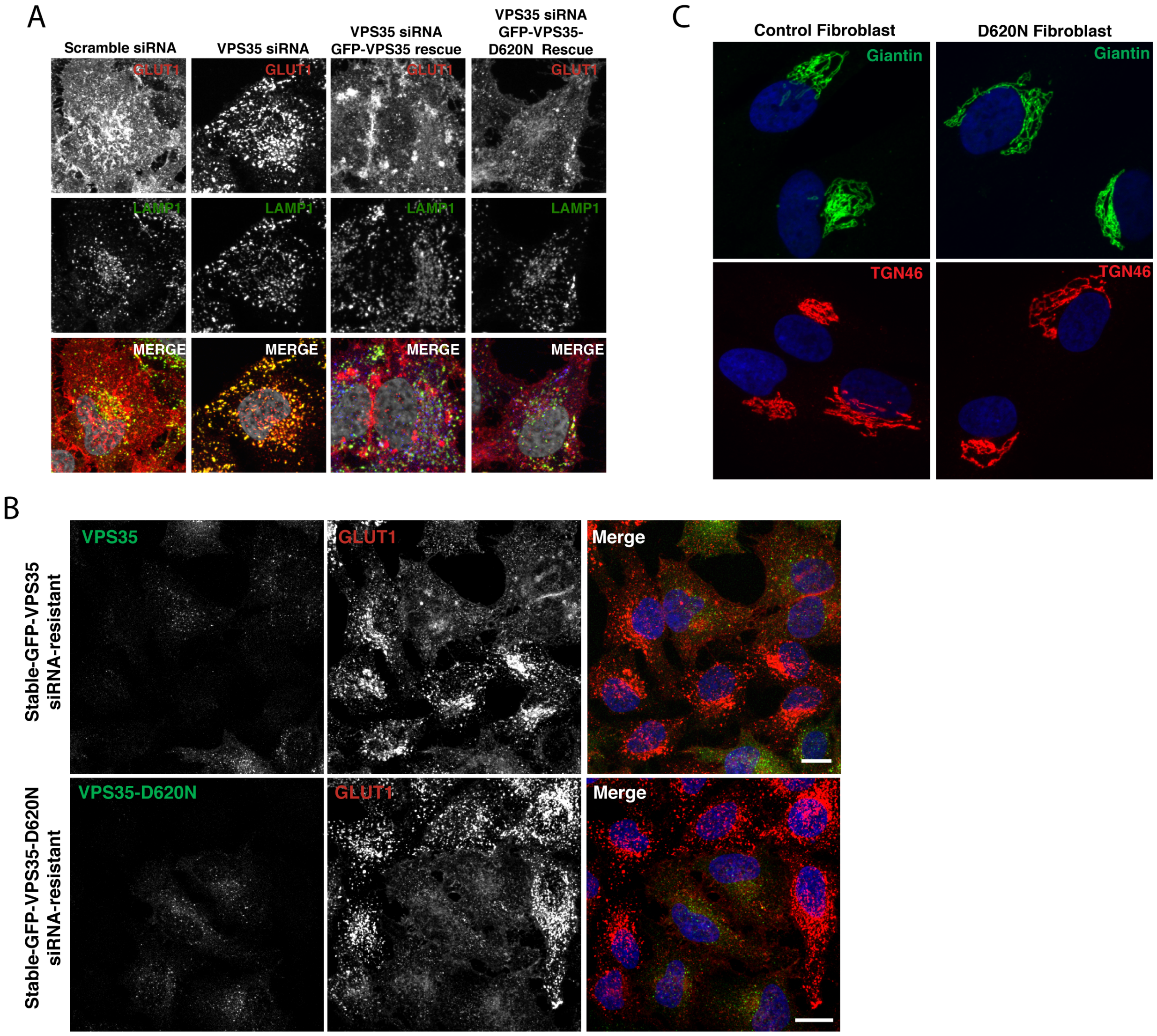

Supplementary Figure 1. The VPS35(D620N) mutation does not impair endosome-to-plasma membrane transport of GLUT1. A: GFP-VPS35(D620N) expression rescues the lysosomal accumulation of GLUT1 caused by VPS35 suppression. HeLa were transfected with non-targeting siRNA or VPS35 siRNA or transduced with lentiviruses encoding for RNAi resistant GFP-VPS35 or GFP-VPS35(D620N) and transfected with siRNA targeting endogenous VPS35. Cells were fixed and stained for VPS35 and GLUT1. Scale bar 10 μm. **B:** The experimental set-up is the same as in A but a larger field of view is shown for side by side comparison of rescue and non rescue cells. **C:** The Golgi is not fragmented in D620N fibroblast cells. The cell lines derived from healthy and D620N patients were fixed and stained for Giantin and TGN46.

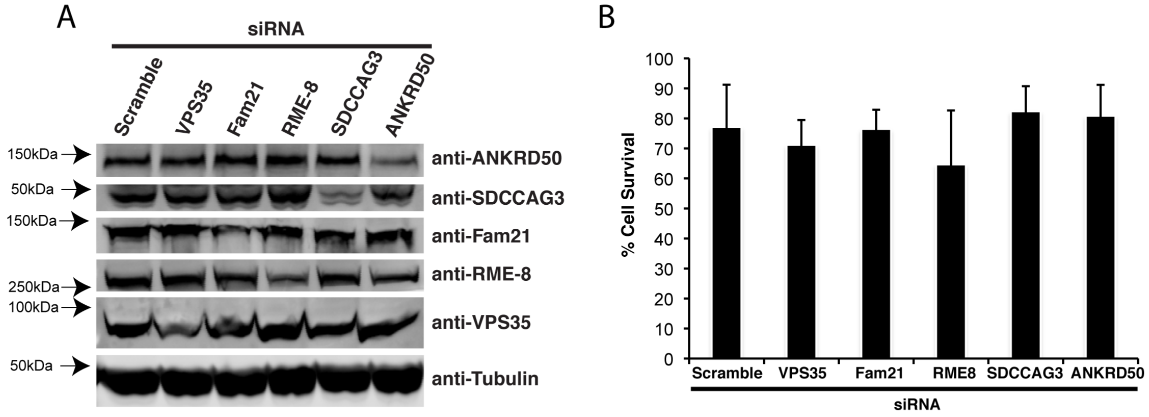

Supplementary Figure 2. Suppression of VPS35 or components identified as being decreased in the GFP-VPS35(D620N) immune-precipitations does not impair cell survival in the SH-SY5Y neuroblastoma cell line. A: SH-SY5Y cells were twice transfected with the indicated siRNAs, the second transfection 48 hours after the first. 24 hours after the second transfection 500 μM of MPP+ was added to the cells. 48 hours after addition of MPP+ cell viability was determined using a 3-(4, 5-dimethylthiazol-2-yl)-5-(3-carboxymethoxyphenyl)-2-(4-sulfophenyl)-2H-tetrazolium salt (MTS) assay according to manufacturer's instructions (Promega).

Supplementary Table 1. SILAC quantified interactome GFP-VPS35 vs. GFP-VPS35(D620N) from human RPE1 cells filtered to include only top heights (the cut-off for filtering was an enrichment of 5 fold or more and with 2 or more peptides quantified). Human RPE1 cells expressing either GFP, GFP-VPS35 or GFP-VPS35(D620N) were cultured in light DMEM (GFP), medium heavy (GFP-VPS35) or in heavy SILAC medium (GFP-VPS35(D620N)), followed by lysis and precipitation with GFP Nanotrap beads. After precipitation, beads were combined, bound proteins resolved by SDS-PAGE and subjected to detection and quantification of heavy and light peptides by LC-MS/MS.

Supplementary Table 2. Unfiltered SILAC quantified interactome GFP-VPS35 vs. GFP-VPS35(D620N) from human RPE1 cells.. The same data set as in Supplementary Table 1 but without the cut-off for enrichment and number of peptides detected. Human RPE1 cells expressing either GFP, GFP-VPS35 or GFP-VPS35(D620N) were cultured in light DMEM (GFP), medium heavy (GFP-VPS35) or in heavy SILAC medium (GFP-VPS35(D620N)), followed by lysis and precipitation with GFP Nanotrap beads. After precipitation, beads were combined, bound proteins resolved by SDS-PAGE and subjected to detection and quantification of heavy and light peptides by LC-MS/MS.

Supplementary Experimental Procedures

Antibodies.

Antibodies used in the study were: rabbit polyclonal WASH1 and FAM21 [15], rabbit polyclonal Strumpellin antibody (Santa Cruz, 87442), rabbit polyclonal VPS26 (Epitomics S1181) and VPS35 (Abcam 97545), mouse monoclonal GFP (clones 7.1/13.1, Roche 11814460001), rabbit polyclonal GLUT1 (Abcam 15309), rabbit monoclonal Itgβ1 (clone EO1042Y, Abcam, 52971), mouse monoclonal TfnR (clone H68.4, Invitrogen 13-6890), rabbit polyclonal LAMP1 (Abcam 24170), rabbit polyclonal RME-8 (gift from D. McPherson, McGill University, Montreal, Canada), rabbit polyclonal Varp (Abcam, ab108216), rabbit polyclonal ANKRD50 (Abcam, ab108219), mouse monoclonal CI-MPR (Abd Serotec, MCA2048T), sheep polyclonal TGN46 (Abd Serotec, AHP500), rabbit polyclonal SDCCAG3 (Proteintech 15969-1-AP), mouse monoclonal anti-β-actin (Applied Biological Materials) and goat anti-VPS35 antibodes (Abcam, 10099).

SILAC interactome analysis.

All SILAC reagents were sourced from Thermo Fisher; except dialysed FBS, which was from Sigma. Human RPE1 cells were grown in the SILAC DMEM for at least 6 passages to achieve full labelling. GFP–VPS35, GFP-VPS35(D620N) and GFP were lentiviral transduced before the labelling. Cells were lysed in precipitation buffer (50 mM Tris–HCl, 0.5% NP40, Roche Protease Inhibitor Cocktail) and GFP was precipitated with GFP-trap beads (Chromotek) for 30 min. Samples were separated on Nupage 4-12% precast gels (Invitrogen) and subjected to LC–MS/MS analysis on an Orbitrap Velos (Thermo) mass spectrometer as previously described [S1, S2].

Cell culture, transfection, immunofluorescence and western blot analysis

HeLa and RPE1 cells were maintained in DMEM (Gibco-Invitrogen) plus 10% (v/v) fetal calf serum (Sigma-Aldrich) and penicillin/streptomycin (PAA). Prior to MPP+ toxicity assays, cells were transfected twice, 48 hours apart, with the indicated siRNA. After the second transfection the cells were incubated in 500 μM MPP+ (Sigma) for 48 hours. Cell viability was determined using a 3-(4, 5-dimethylthiazol-2-yl)-5-(3-carboxymethoxyphenyl)-2-(4-sulfophenyl)-2H-tetrazolium salt (MTS) assay according to manufacturer's instruction (Promega). For immunofluorescence analysis, cells were fixed in 0.1 M phosphate buffer containing 4% (w/v) paraformaldehyde for 10 minutes on ice and permeabilized with 0.1% (v/v) Triton X-100 for 5 minutes. Thereafter, cells were incubated with 0.5% (w/v) BSA for 30 minutes followed by incubation with the indicated primary antibodies and subsequent incubation with secondary antibodies (Molecular Probes). For nuclear staining DAPI was used. Images were recorded on a Leica SPE or a Leica AOBS-SP2 confocal microscope. Following acquisition, images were analysed with the Volocity software package (Perkin Elmer): to filter noise, thresholds were applied uniformly across conditions. After setting of the thresholds, the percentage of co-localization and the Pearson's correlation between the respective channels were quantified with the co-localization tool of the Volocity software. Each co-localization analysis is based on the quantification of at least 150 images. For immuno-precipitation experiments the respective cell lines stably transduced with the desired GFP-tagged constructs were lysed in Tris-based immunoprecipitation buffer (50 mM Tris–HCl, 0.5% NP40 and Roche Protease inhibitor cocktail) and GFP precipitated with GFP-trap beads (Chromotek). Immuno-blottling was performed using standard procedures. Detection was carried out on a Licor Odyssey Infrared scanning system using fluorescently labelled secondary antibodies.

siRNA.

For VPS35 suppression the on-target plus human smartpool (Dharmacon) was used, except when suppressed in cells stably expressing a GFP-VPS35 siRNA resistant construct, in which case only oligo-3 from the smartpool was used. RME-8, ANKRD50 and FAM21 were suppressed using on-target plus human smartpools (Dharmacon). SDCCAG3 was suppressed using the following oligo: AAU UCU AAG CUG AGA AGA ATT.

Qantification of the surface levels of GLUT1

For the quantification of protein surface, cells were surface biotinylated with a commercially available kit (Pierce/Thermo) according to the manufacturer's instructions seventy-two hours post transfection. Cells were lysed in PBS with 2% Triton X-100, lysates were pooled and streptavidin agarose (GE-Healthcare) was used to capture biotinylated membrane proteins. After capture, biotinylated proteins were washed extensively in 1.2 M NaCl containing PBS with 1% Triton X-100 to remove cytoskeletal proteins and contaminants from the transmembrane proteins, followed by elution of the proteins by boiling in dithiothreitol-containing sample buffer.

Degradation assays.

To measure degradation of surface proteins, HeLa cells were transfected with the requisite siRNA. Twenty-four hours post transfection, before the effects of the siRNA were fully established, surface proteins were biotinylated with sulpho-NHS-biotin (Pierce/Thermo). Zero, twelve and twenty-four hours after biotinylation (24, 36 and 48 hours post transfection of siRNA), cells were lysed, biotinylated proteins were

captured with streptavidin agarose (GE-Healthcare) and detected by quantitative western blotting on an Odyssey scanner. Degradation was quantified as fluorescence signal remaining after 12 and 24 hours in percentage of signal intensity at time point 0.

VPS35 constructs and mutagenesis.

VPS35 was subcloned into pXLG3. shRNA viral construct against the 3'UTR of VPS35 was purchased from Sigma (TRCN0000337019). The D620N mutation (1858G>A) was generated using site-directed mutagenesis with the following primer 5'-GTA TGA AGA TGA AAT CAG CAA TTC CAA AGC-3'. siRNA resistant VPS35 was generated by introducing 6 silent base mis-matches (T1314A, T1317C, G1320A, C1321T, T1323A, T1326C) into the open reading frame, conferring resistance to siRNA VPS35-3 as described above.

Protein purification and isothermal titration calorimetry (ITC):

Protein purification and the ITC experiments were performed as previously described [S1, S2]. Briefly, equal amounts of BL21 (DE3) T1R cells (New England Biolabs, NEB) individually expressing GST-fusions of full-length VPS35 and VPS29 were mixed and co-lysed. The formed VPS35/VPS29 dimers were purified by glutathione-sepharose 4B affinity beads (GE Healthcare) and treated by TEV protease to remove the GST tag. The cleaved proteins were further purified by anion exchange (SOURCE 15Q, GE Healthcare) and size exclusion (Superdex 200, GE Healthcare) chromatographies. FAM21 fragments were expressed with a N-terminal TEV-cleavable MBP tag and a C-terminal, noncleavable His6 tag. The FAM21 proteins were purified by Amylose resin (NEB), subjected to TEV protease digestion, and by Ni-NTA resins (Qiagen). The purified VPS35/29 complex and FAM21 fragments were dialyzed in the same ITC buffer

(20 mM Tris, pH 8.0, 100 mM NaCl and 5 mM β-mercaptoethanol) for at least 24 hours at 4 °C. The ITC experiments were performed using a VP-ITC microcalorimeter (Microcal) at 20 °C by titrating 100 – 200 μM FAM21 proteins into 10 – 16 μM VPS35/VPS29 complex. Heat peaks were detected and integrated using NITPIC [S3]. The binding isotherms were globally fitted using SEDPHAT [S4]. Since the R20-21 fragment contains two binding sites for the VPS35/VPS29 complex, a 'two-site heterogeneous association (A+B+B=ABB)' model was chosen for fitting using the following variables: inclA (fraction of FAM21 incompetent for binding), log(Ka1) (logarithm of the macroscopic constant for the first binding), HAB (enthalpy change for the first binding). For each protein complex, the binding constants and thermodynamic parameters from three independent experiments were subjected to global fitting. We also tried to fit the ITC data with two different values for both Kd and enthalpy change, but could not obtain a statistically better fit. Finally, the limited amount of the data prevents us from analysing and reporting cooperativity.

Derivation of human VPS35(D620N) dermal fibroblasts from a skin biopsy:

Two 2 mm^2 skin biopsies were taken from the forearm of a healthy donor patient and a Parkinson's disease patient genotype harboring the VPS35(D620N) mutation. Each biopsy was placed into a sterile sample tube (Sterilin, UK) with approximately 10 ml of Fibroblast media. The samples were transported to the lab on wet ice in < 30 mins to be processed. In a class II hood, a sterile scalpel and forceps were used to remove the epidermis and any fat from the tissue and then cut into 4 pieces. These pieces are then placed into a 4 well plate (Nunc) pre-coated with CELLstart™ ((1:200 in PBS(+/+)), Sigma D8662) in 200 µl of Fibroblast media (DMEM (Sigma, D6546) supplemented with 10% Foetal Bovine Serum (Heat Inactivated), (Invitrogen, 10500), 1% Glutamax™ (Invitrogen, 35050), 1% NEAA (Invitrogen, 11140), 1% Penicillin-Streptomycin (Sigma,

P4458). The pieces were left to sit up to two weeks until they were attached to the matrix and the dermal fibroblasts started to proliferate. The cells were left to 80% confluency before being enzymatically passaged using TrypLE Express (Gibco, 12604). The cells were passaged at a ratio of 1:3 until cryopreserved.

Supplementary References

S1. Steinberg, F., Gallon, M., Winfield, M., Thomas, E., Bell, A.J., Heesom, K.J., Tavare, J.M., and Cullen, P.J. (2013). A global analysis of SNX27-retromer assembly and cargo specificity reveals a function in glucose and metal ion transport. Nat. Cell Biol. *15*, 461-471.

S2. Steinberg, F., Heesom, K.J., Bass, M.D., and Cullen, P.J. (2012). SNX17 protects integrins from degradation by sorting between lysosomal and recycling pathways. J. Cell Biol. *197*, 219-230.

S3. Keller, S., Vargas, C., Zhao, H., Piszczek, G., Brautigam, C.A., and Schuck, P. (2012). High-precision isothermal titration calorimetry with automated peak-shape analysis. Anal. Chem. *84*, 5066-5073.

S4. Houtman, J.C., Brown, P.H., Bowden, B., Yamaguchi, H., Appella, E., Samelson, L.E., and Schuck, P. (2007). Studying multisite binary and ternary protein interactions by global analysis of isothermal titration calorimetry data in SEDPHAT: application to adaptor protein complexes in cell signaling. Protein Sci. *16*, 30-42.
