# Supplementary material for: Retromer Binding to FAM21 and the WASH Complex Is Perturbed by the Parkinson Disease-Linked VPS35(D620N) Mutation
Source: Curr Biol. 2014 Jul 21;24(14):1670–6. doi: 10.1016/j.cub.2014.06.024 (PMC4110399; doi:10.1016/j.cub.2014.06.024)
Supplement: Document S2. Article plus Supplemental Information [file mmc4.pdf]

Current Biology 24, 1670–1676, July 21, 2014 ©2014 The Authors http://dx.doi.org/10.1016/j.cub.2014.06.024

Report

Retromer Binding to FAM21 and the WASH Complex Is Perturbed by the Parkinson Disease-Linked VPS35(D620N) Mutation

Ian J. McGough,[1] Florian Steinberg,[1] Da Jia,[2]
Peter A. Barbuti,[3] Kirsty J. McMillan,[1] Kate J. Heesom,[4]
Alan L. Whone,[5] Maeve A. Caldwell,[3] Daniel D. Billadeau,[6]
Michael K. Rosen,[2,7] and Peter J. Cullen[1,*]
[1]The Henry Wellcome Integrated Signaling Laboratories, School of Biochemistry, Medical Sciences Building, University of Bristol, Bristol BS8 1TD, UK
[2]Department of Biophysics, University of Texas Southwestern Medical Center, Dallas, TX 75390, USA
[3]Henry Wellcome Laboratory for Integrative Neuroscience and Endocrinology, School of Clinical Sciences, University of Bristol, Dorothy Hodgkin Building, Whitson Street, Bristol BS1 3NY, UK
[4]Proteomics Facility, School of Biochemistry, Medical Sciences Building, University of Bristol, Bristol BS8 1TD, UK
[5]Institute of Clinical Neurosciences, University of Bristol, Frenchay Hospital, Bristol BS16 1LE, UK
[6]Departments of Biochemistry and Molecular Biology and Immunology, Mayo Clinic, Rochester, MN 55905, USA
[7]Howard Hughes Medical Institute, University of Texas Southwestern Medical Center, Dallas, TX 75390, USA

Summary

Retromer is a protein assembly that plays a central role in orchestrating export of transmembrane-spanning cargo proteins from endosomes into retrieval pathways destined for the Golgi apparatus and the plasma membrane [1]. Recently, a specific mutation in the retromer component VPS35, VPS35(D620N), has linked retromer dysfunction to familial autosomal dominant and sporadic Parkinson disease [2, 3]. However, the effect of this mutation on retromer function remains poorly characterized. Here we established that in cells expressing VPS35(D620N) there is a perturbation in endosome-to-TGN transport but not endosome-to-plasma membrane recycling, which we confirm in patient cells harboring the VPS35(D620N) mutation. Through comparative stable isotope labeling by amino acids in cell culture (SILAC)-based analysis of wild-type VPS35 versus the VPS35(D620N) mutant interactomes, we establish that the major defect of the D620N mutation lies in the association to the actin-nucleating Wiskott-Aldrich syndrome and SCAR homolog (WASH) complex. Moreover, using isothermal calorimetry, we establish that the primary defect of the VPS35(D620N) mutant is a 2.2 ± 0.5-fold decrease in affinity for the WASH complex component FAM21. These data define the primary molecular defect in retromer assembly that arises from the VPS35(D620N) mutation and, by revealing functional effects on retromer-mediated endosome-to-TGN transport, provide new insight into retromer deregulation in Parkinson disease.

*Correspondence: pete.cullen@bristol.ac.uk
This is an open access article under the CC BY license (http://creativecommons.org/licenses/by/3.0/).

Results and Discussion

Endosome-to-TGN Transport of CI-MPR Is Impaired in VPS35(D620N)-Expressing Cells

We sought to investigate the effect of the VPS35(D620N) mutation on trafficking of two known retromer cargos, the endosome-to-TGN transport of the cation-independent mannose 6-phosphate receptor (CI-MPR) [4–7], and the endosome-to-plasma membrane transport of the glucose transporter GLUT1 [8]. At steady state, GLUT1 is localized at the plasma membrane from where it undergoes continuous rounds of endocytosis and PDZ ligand-dependent endosome-to-plasma membrane recycling [9], the latter being mediated by the SNX27-retromer [8]. In the absence of retromer, GLUT1 accumulates in the lysosome and is degraded [8]. To establish whether retromer-mediated endosome-to-plasma membrane transport was affected by the VPS35(D620N) mutation, we performed a quantitative analysis of GLUT1 surface abundance, lysosomal localization, and kinetics of lysosomal-mediated degradation [8]. In HeLa or RPE1 cells, the depletion of endogenous VPS35 by siRNA-mediated suppression followed by re-expression of either wild-type GFP-VPS35 or GFP-VPS35(D620N) produced cell lines where the GFP-tagged VPS35 transgenes were expressed at near to endogenous levels (Figure 1C). In these cells, expression of GFP-VPS35 or GFP-VPS35(D620N) efficiently rescued the lysosomal missorting of GLUT1 observed upon VPS35 suppression (Figures 1A and 1B) (see Figure S1A available online for split channels and Figure S1B for a larger field of view). Furthermore, while the knockdown of VPS35 in RPE1 cells led to a pronounced decrease of GLUT1 surface abundance, re-expression of wild-type or mutant VPS35 rescued GLUT1 surface abundance (Figures 1A and 1C). Finally, an analysis of GLUT1 degradation kinetics in the RPE1 cells revealed that the post-translational stability of GLUT1 was not affected by the VPS35(D620N) mutation (Figures 1Di–1Diii): the degradation of transferrin receptor was also monitored as a negative control and as expected its degradation rate was also unaffected by the VPS35(D620N) mutation. Overall, these data establish that the VPS35(D620N) mutation does not impair retromer-mediated endosome-to-plasma membrane transport of GLUT1.

Next, we examined the endosome-to-TGN transport of the CI-MPR. After delivery to endosomes, CI-MPR dissociates from its ligand and is recognized by the retromer complex and retrieved to the TGN for further rounds of ligand binding and transport [4–7]. In the absence of retromer, the efficiency of CI-MPR retrieval is perturbed and an increase in endosomal localization of CI-MPR is observed [4–7]. At steady state in our RPE1 cell line, CI-MPR was predominantly localized to the TGN (Figure 1Ei). Suppression of VPS35 led to an increase in the amount of CI-MPR on dispersed puncta and a decrease in the Pearson's correlation between CI-MPR and TGN46 (Figures 1Ei and 1F). This dispersal and decrease in Pearson's correlation was partially rescued by re-expressing GFP-VPS35 but not GFP-VPS35(D620N) (Figures 1Ei and 1F). The punctate CI-MPR staining in the VPS35(D620N)-expressing

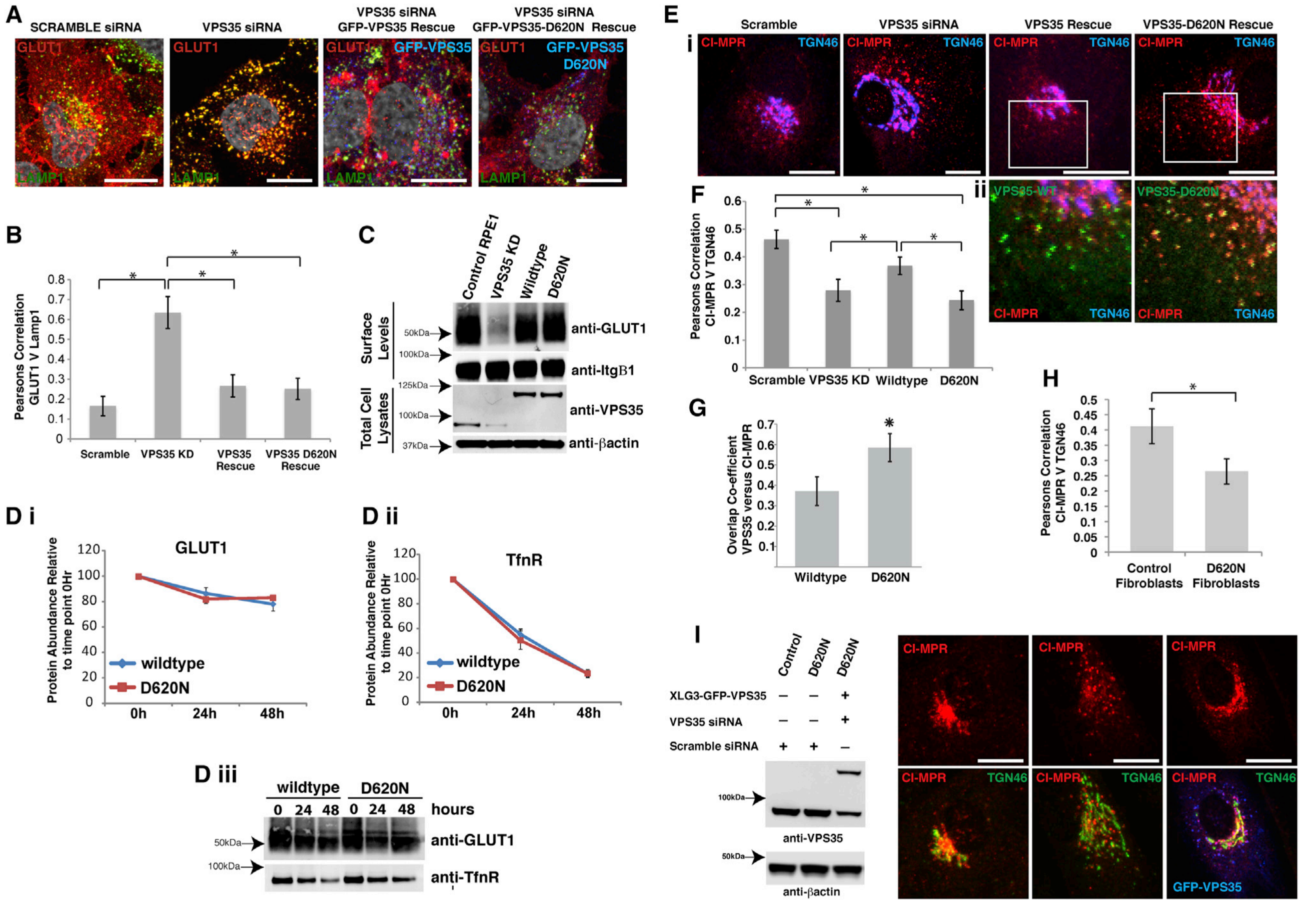

Figure 1. The VPS35(D620N) Mutation Impairs Endosome-to-TGN Transport of CI-MPR

(A) GFP-VPS35(D620N) expression rescues the lysosomal accumulation of GLUT1 caused by VPS35 suppression. HeLa cells were transduced with lentiviruses encoding for RNAi-resistant GFP-VPS35 or GFP-VPS35(D620N) and transfected with siRNA-targeting endogenous VPS35 or a nontargeting siRNA. Cells were fixed and stained for VPS35, LAMP1, and GLUT1. Scale bar, 10 μm.

(B) The Pearson's correlation between GLUT1 and Lamp1, in the conditions outlined in (A), quantified from three independent experiments. Approximately 45 cells were analyzed per experiment (*$p < 0.01$; one-way ANOVA followed by post hoc Tukey test; error bars, SEM).

(C) GFP-VPS35(D620N) expression rescues the loss of GLUT1 from the cell surface caused by VPS35 suppression. The surface abundance of GLUT1 in the indicated stable RPE1 cells was determined by quantitative immunoblotting.

(Di–Diii) GLUT1 and TfnR degradation kinetics are unaffected by the VPS35(D620N) mutation. The surface of GFP-VPS35 and GFP-VPS35(D620N) cells were randomly biotinylated. Biotinylated proteins were captured from cell lysates with streptavidin beads at indicated time points after biotinylation and subjected to quantitative immunoblotting on an Odyssey scanner. The plots represent the mean of nine independent experiments (error bars, SEM).

(Ei, Eii, and F) Steady-state localization of endogenous CI-MPR is perturbed in GFP-VPS35(D620N) RPE1 cells. RPE1 cells suppressed for VPS35 expression and rescued through stable expression of GFP-VPS35 or GFP-VPS35(D620N) were fixed and stained for endogenous CI-MPR (red) and TGN46 (blue). Scale bar, 10 μm.

(F) The Pearson's correlation between CI-MPR and TGN46 from three independent experiments (approximately 45 cells were analyzed per experiment) is shown (*$p < 0.01$; one-way ANOVA followed by post hoc Tukey test; error bars, SEM).

(G) The percentage of overlap between VPS35 or VPS35(D620N) and CI-MPR in the cell periphery (entire cell area excluding the TGN region) is shown in bar graph format. Approximately 20 cells from three independent experiments were analyzed (*$p < 0.05$; unpaired t test; error bars, SD).

(H) Fibroblasts from a healthy donor and a patient harboring the VPS35(D620N) mutation were fixed and stained for endogenous CI-MPR and TGN46. The Pearson's correlation between CIMPR and TGN46 from three independent experiments (approximately 35 cells were analyzed per experiment) is shown (*$p < 0.05$; unpaired t test; error bars, SEM).

(I) Patient-derived VPS35(D620N) fibroblasts suppressed for endogenous VPS35 and re-expressing wild-type GFP-VPS35 were fixed and stained for endogenous CI-MPR and TGN46. Scale bar, 10 μm.

cells was positive for VPS35 (Figure 1Eii), and there was an increase in the overlap between CI-MPR and VPS35 in the GFP-VPS35(D620N) cells when compared to GFP-VPS35-expressing cells (Figure 1G), consistent with a defect in endosome-to-TGN transport and a corresponding CI-MPR dispersal. To extend this, we also examined the steady-state distribution of CI-MPR in fibroblasts obtained from a healthy donor and from a patient harboring the VPS35(D620N) mutation. Again an increased dispersal of CI-MPR was observed (Figures 1H and 1I). This dispersal was not due to a fragmented Golgi as both TGN46 and GRASP65 distribution appeared normal in VPS35(D620N) fibroblasts (Figure S1C). The CI-MPR dispersal observed in the VPS35(D620N) fibroblasts could be rescued by suppression of endogenous VPS35 and re-expression of wild-type GFP-VPS35 (Figure 1I), suggesting that the differences in CI-MPR distribution are not due to variability between cell lines isolated from different patients. These data confirm and extend recent findings of an impairment in

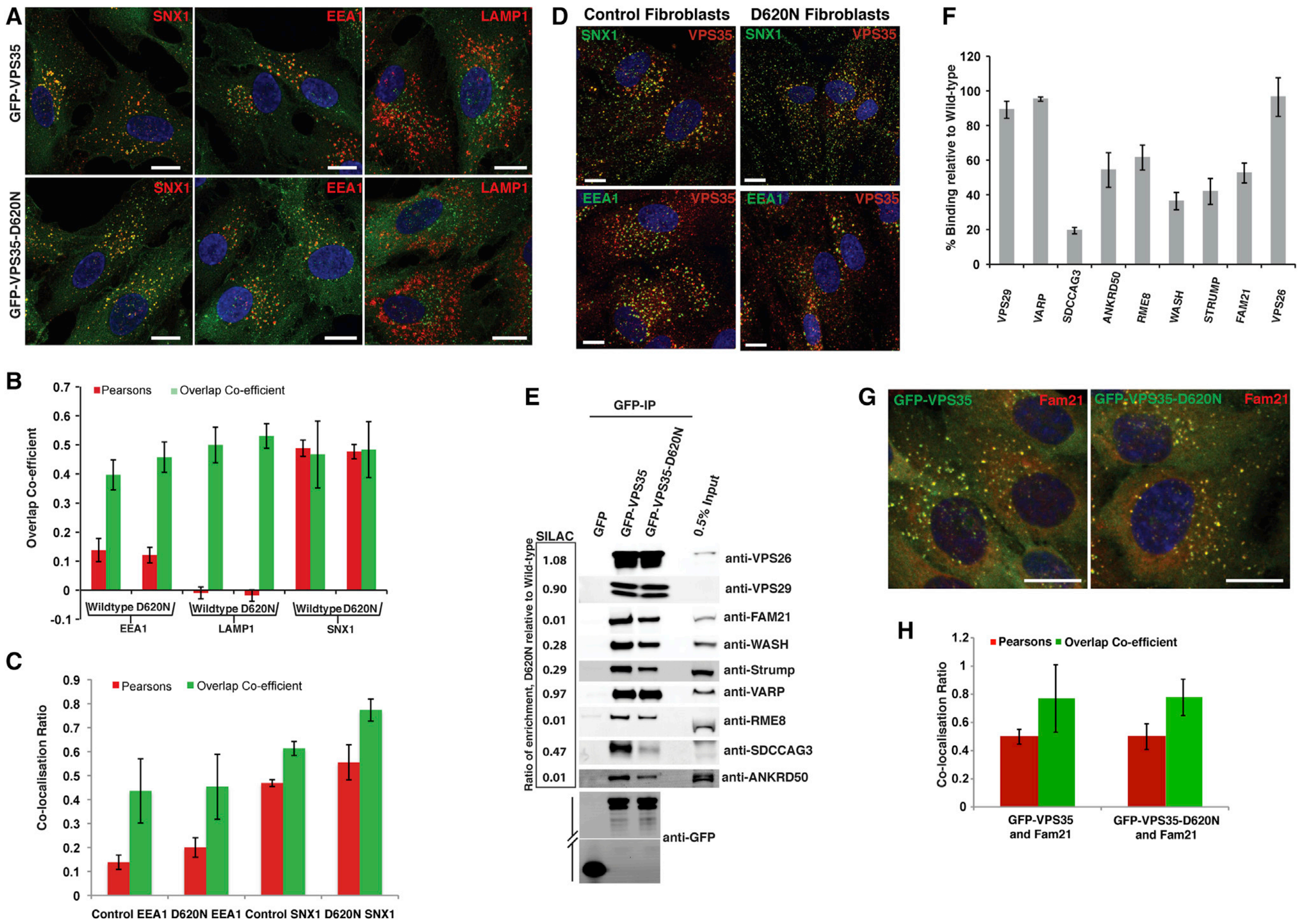

Figure 2. The D620N Mutation in VPS35 Results in a Decrease in Association with the WASH Complex

(A and B) Endosomal localization of VPS35 is not perturbed by the VPS35(D620N) substitution. GFP-VPS35- and GFP-VPS35(D620N)-expressing cells were fixed and stained for SNX1, EEA1, and LAMP1 (A). The percentage of overlap and Pearson's correlation are shown in bar graph format (15 fields of view [approximately 150 cells] from three independent experiments were analyzed; error bars, SEM) (B). Scale bar, 10 μm.

(C and D) Fibroblasts from a healthy donor and a patient harboring the VPS35(D620N) mutation were fixed and stained for endogenous SNX1, EEA1, and VPS35 (D). The percentage overlap and Pearson's correlation are shown in bar graph format (approximately 40 cells from three independent experiments were analyzed; error bars, SEM) (C). Scale bar 10 μm.

(E) GFP-VPS35(D620N) immunoprecipitates WASH complex components to a lesser extent than wild-type VPS35. SILAC-based comparative proteomic analysis of GFP-trap-precipitated GFP versus GFP-VPS35 and GFP-VPS35(D620N). The GFP-VPS35, GFP-VPS35(D620N), and GFP stable RPE1 cell lines were grown in normal (GFP), medium-isotope-labeled (VPS35) medium, and heavy-isotope-labeled (VPS35(D620N)) medium, followed by lysis and precipitation with GFP-trap beads. Precipitates were pooled, separated by SDS-PAGE, and subjected to gel walking LC–MS/MS analysis on an Orbitrap mass spectrometer. The SILAC ratio is the fold-enrichment of proteins in GFP-VPS35(D620N) precipitates over GFP-VPS35. Quantitative fluorescent-based immunoblot analysis of precipitates from the stable RPE1 cell lines broadly confirmed and correlated with the SILAC data.

(F) The immunoprecipitates were repeated three times and the mean fluorescent intensity of selected interactors in the GFP-VPS35(D620N) immunoprecipitate compared to the corresponding intensity in the GFP-VPS35 immunoprecipitate are shown (error bars, SEM).

(G and H) Endosomal localization of FAM21 is not perturbed by the VPS35(D620N) substitution. GFP-VPS35- and GFP-VPS35(D620N)-expressing cells were fixed and stained for FAM21 (G). The Pearson's correlation and overlap coefficient are shown in bar graph format (40 cells) from three independent experiments were analyzed (error bars, SEM) (H).

retromer-mediated endosome-to-TGN transport in cells carrying the VPS35(D620N) mutation [10, 11].

The VPS35(D620N) Mutant Does Not Perturb the Formation of the Retromer Heterotrimer or the Endosome Localization of VPS35

To determine the molecular basis of the perturbed endosome-to-TGN trafficking observed with the VPS35(D620N) mutant, we first sought to determine whether the VPS35(D620N) mutation affected formation of the VPS26-VPS29-VPS35 heterotrimer or its endosomal localization. Like GFP-VPS35, GFP-VPS35(D620N) localized predominantly to a sorting nexin-1 (SNX1) positive early-to-late transitional endosomal compartment (Figures 2A and 2B). In agreement with this, VPS35 showed normal distribution and appearance of the retromer-decorated endosomal compartment in the fibroblast cells derived from patient samples as well as comparable colocalization with SNX1 and the early endocytic marker EEA1 (Figures 2C and 2D). Immunoisolation followed by western blotting also established that VPS35(D620N) retained the ability to form a heterotrimeric complex with VPS29 and VPS26A (Figure 2E). These data establish that the molecular defect associated with the VPS35(D620N) mutation lies outside of heterotrimeric retromer assembly and retromer's endosome association.

Comparative Interactome Analysis of VPS35 and the VPS35(D620N) Mutant

Retromer scaffolds the assembly of a number of accessory proteins that aid its role in endosomal sorting [1]. To identify the effect of the VPS35(D620N) mutant on the assembly of these accessory proteins, we performed an unbiased quantitative analysis of the VPS35 and VPS35(D620N) interactomes. In a triple SILAC setup, RPE1 cell lines stably expressing GFP, GFP-VPS35 or GFP-VPS35(D620N) were grown in light (GFP), medium heavy (GFP-VPS35) and heavy (GFP-VPS35(D620N)) SILAC media until full steady-state labeling was ensured. Cells were then subjected to lysis, GFP trap immunoisolation and pooling of the samples for SDS-PAGE, and subsequent quantification by LC-MS/MS after in-gel tryptic digestion. Known VPS35-interacting partners VPS26A and VPS26B, VPS29, TBC1D5 [12], SNX27 [8], and all components of the actin-polymerizing Wiskott-Aldrich syndrome and SCAR homolog (WASH) complex [12–17] were enriched in the wild-type GFP-VPS35 immunoisolation (Tables S1 and S2). In addition, a number of previously unidentified interactors including VARP, SDCCAG3, and ANKRD50 were identified, the functional relevance of which will be described elsewhere.

While there is comparative enrichment of VPS29, VPS26A, and VPS26B between wild-type and VPS35(D620N) (left side of Figure 2E; Tables S1 and S2), there was a clear decrease in the enrichment of all components of the WASH complex within the VPS35(D620N) interactome (Figure 2E; Tables S1 and S2). In addition, there was a decrease in enrichment of the known retromer interactor RME-8 [18, 19] and two of the newly identified interactors SDCCAG3 and ANKRD50 (Figure 2E; Tables S1 and S2). In contrast, there was no loss in binding to other known retromer interactors such as TBC1D5 [20] and SNX27 [8] and the newly identified interactor VARP [12] (Figure 2E; Tables S1 and S2). Validating the mass spectrometric quantification, fluorescence-based quantitative western analysis confirmed the decrease in binding of these proteins to the VPS35(D620N) mutant (Figures 2E and 2F). Given the loss of binding to the WASH complex observed in the GFP-VPS35(D620N) cells, we sought to investigate whether the membrane association of the WASH complex is impaired in the VPS35(D620N)-expressing cells. We saw no obvious impairment of FAM21 endosomal localization or a decrease in Pearson's correlation between GFP-VPS35(D620N) and FAM21 (Figures 2G and 2H). However, we cannot rule out that the loss of binding to the WASH complex alters turnover of the WASH complex on the endosome or affects the endosomal subdomain organization of the retromer and WASH complexes.

The Binding of ANKRD50, SDCCAG3, and RME-8 to Retromer Is Mediated through the WASH Complex

To identify the primary defect in the VPS35(D620N) mutant, we performed a series of GFP-VPS35 immunoisolations from cells treated with RNAi's targeting FAM21—the component of the WASH complex that directly binds to VPS35 [16], ANKRD50, SDCCAG3, and RME-8. Suppression of FAM21 resulted in a loss in the enrichment of ANKRD50, SDCCAG3, and RME-8 in GFP-VPS35 immunoisolates (Figures 3A–3C), establishing that these proteins interact indirectly with retromer through the WASH complex. Suppression of ANKRD50, SDCCAG3, and RME-8 did not affect the binding of FAM21 to VPS35 (Figures 3A–3C).

To correlate these data with the previously described endosome association of SDCCAG3 and RME-8 [21, 22], we analyzed parallel experiments using immunofluorescence. Confirming published data, suppression of VPS35 led to the dissociation of FAM21 from endosomes (Figures 3D and 3E) [15, 16]. Consistent with the biochemical analysis, the endosome association of SDCCAG3 required the presence of both retromer and FAM21 (Figures 3D and 3E). In contrast, the endosome association of RME-8 was only partially dependent on retromer and FAM21 (Figures 3D and 3E), which is consistent with the known binding of RME-8 to other endosome-associated proteins including SNX1 [18]. Together, these data establish that the association of ANKRD50, SDCCAG3, and RME-8 to retromer is mediated through binding to the WASH complex, and for SDCCAG3 this is necessary for targeting to retromer-labeled endosomes. Moreover, they provide evidence that the major defect of the VPS35(D620N) mutant lies with the association to the WASH complex.

The VPS35(D620N) mutation has been suggested to inhibit the neuroprotective effect of VPS35 [23], so we sought to investigate whether suppression of VPS35, FAM21, SDCCAG3, RME-8, or ANKRD50 enhanced the toxicity and increased cell death observed in the neuroblastoma cell line SH-SY5Y upon exposure to MPP+, which interferes with complex I of the electron transport chain in mitochondrion, causing cell death. However, no increase in cell death upon suppression of any of these components was observed (Figure S2) when the cells were incubated in 500 μM of MPP+ for 48 hr. This is in line with a previous study that found no effect of VPS35 suppression on cell survival when SH-SY5Y cells were challenged with MPP+ [23]. However, the absence of an effect could also be due to insufficient suppression of these components in SH-SY5Y cells.

VPS35(D620N) Impairs Its Interaction with FAM21 In Vitro

Previous studies have suggested that the interaction between the retromer and FAM21 is mediated by the VPS35 and VPS29 (but not VPS26) subunits of the retromer heterotrimer and multiple LFa motifs from the C terminus of FAM21 [15, 16, 24]. FAM21 contains 21 such LFa motifs with varied affinities to the VPS35/VPS29 complex. The two most C-terminal ones, R20 and R21, bind to the VPS35/VPS29 complex with the highest affinity, and deletion of R20-21 abolished endosomal localization of FAM21 in cells [16]. Thus, to evaluate the effect of the VPS35(D620N) substitution, we examined whether this mutation affects the affinity of the VPS35/VPS29 complex to the R20-21 fragment by using isothermal titration calorimetry (ITC).

The recombinant VPS35/VPS29 complex was purified by colysing individually expressing bacterial cells as described previously [16]. The VPS35(D620N) substitution did not affect complex formation with VPS29, further confirming our biochemical and proteomic analysis (Figures 2E and 2F). The ITC experiments for each VPS35/VPS29 complex were repeated three times with two independently prepared protein samples and slightly different injection protocols. Raw data were pooled and subjected to global fitting using the software package SEDPHAT. Similar to our previous studies, VPS35/VPS29 bound R20-21 with a stoichiometry of 1:2 [16]. The affinity of VPS35(D620N)/VPS29 to R20-21 (K_d = 4.6 ± 0.7 μM) was significantly lower (by 2.2 ± 0.5-fold) than that of the wild-type VPS35/VPS29 (K_d = 2.4 ± 0.6 μM) (Figures 4A and 4B). These results establish that the VPS35(D620N) substitution impairs its interaction with FAM21.

In addition to R20-21, we also attempted to measure the affinity for a larger fragment of FAM21, R15-21. Although the ITC thermograms were qualitatively consistent with a reduced

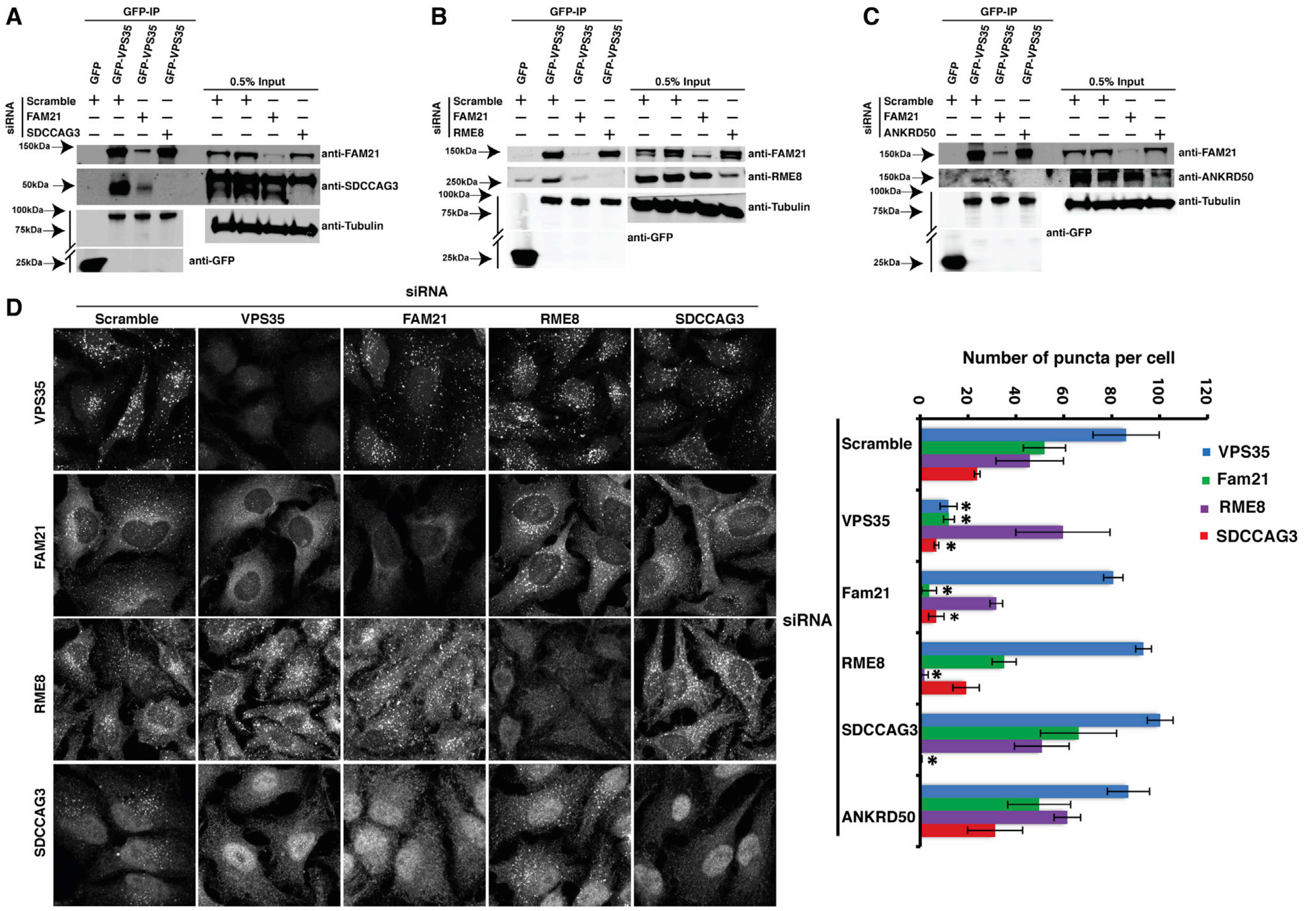

Figure 3. The WASH Complex Is Required for the Association of RME8, SDCCAG3, and ANKRD50 with VPS35

(A, B, and C) GFP-VPS35-expressing RPE1 cells were transfected with nontargeting siRNA or siRNA against FAM21, RME-8, SDCCAG3, or ANKRD50 prior to GFP-trap immunoprecipitation of GFP-VPS35 and immunoblotting with antibodies raised against FAM21, SDCCAG3, RME-8, ANKRD50, Tubulin, and GFP. Suppression of FAM21 resulted in a loss of SDCCAG3 (A), RME-8 (B), and ANKRD50 (C) from VPS35 immunoprecipitates. Suppression of RME-8, SDCCAG3, or ANKRD50 did not affect the ability of VPS35 to immunoprecipitate FAM21.

(D) FAM21 and retromer are required for the endosomal localization of SDCCAG3. HeLa cells were transfected with nontargeting siRNA or siRNA against VPS35, FAM21, RME-8, or SDCCAG3 prior to imaging of the endogenous localization of each protein. Scale bar, 10 μm.

(E) The number of puncta per cell above a set threshold intensity, from three independent experiments (30 cells per experiment), for the indicated proteins was quantified using Volocity (*p < 0.01; one-way ANOVA followed by post hoc Tukey test; error bars, SD).

affinity for the VPS35(D620N) mutant, since the R15-21 fragment contains seven LFa motifs potentially interacting with VPS35/VPS29, it was not possible to quantitatively fit and interpret these data (data not shown).

In summary, stemming from an unbiased quantitative proteomic analysis, we have identified that at the molecular level the primary effect of the Parkinson disease-linked VPS35(D620N) mutation is a decrease in the affinity for FAM21 and as a consequence a reduced association with the WASH complex and its associated proteins, including RME-8, and the newly identified retromer-associated proteins SDCCAG3 and ANKRD50. While not perturbing retromer-mediated endosome-to-plasma membrane recycling of GLUT1, this partial loss of FAM21 binding and the WASH complex-associated proteins does lead to a decrease in the efficiency of endosomes-to-TGN retrieval of the CI-MPR. While the primary defect of the VPS35(D620N) mutation is a decrease in affinity for the WASH complex, it is important to stress that the observed phenotypes maybe arise from a reduced activity of RME8, SDCCAG3, or ANKRD50 or a combination of these and FAM21. This is consistent with the current view that WASH-mediated actin polymerization is required for the organization and maturation of retromer sorting domains on early-to-late transition endosomes [16]. Precisely why the decrease in coupling to the WASH complex, and associated proteins, leads to a selective retromer effect on endosome-to-TGN over endosome-to-plasma membrane transport is unclear and will require further investigation. With evidence linking mutations in the WASH complex component strumpellin to hereditary spastic paraplegia [25], and the recent description of an RME-8(N855S) mutation that segregates with late-onset Parkinson disease [26], the data presented here establish that the association and function relationship between retromer and WASH complexes will be an important area in defining the role of defective endosomal sorting in these neurodegenerative diseases.

During the final stages of reviewing our manuscript Zavodszky and colleagues, using a candidate approach, published work consistent with a defect in WASH binding to the VPS35(D620N) mutant [27]. Our detailed biochemical analysis extends their findings and, importantly, through our unbiased global analysis, highlights that the corresponding loss of

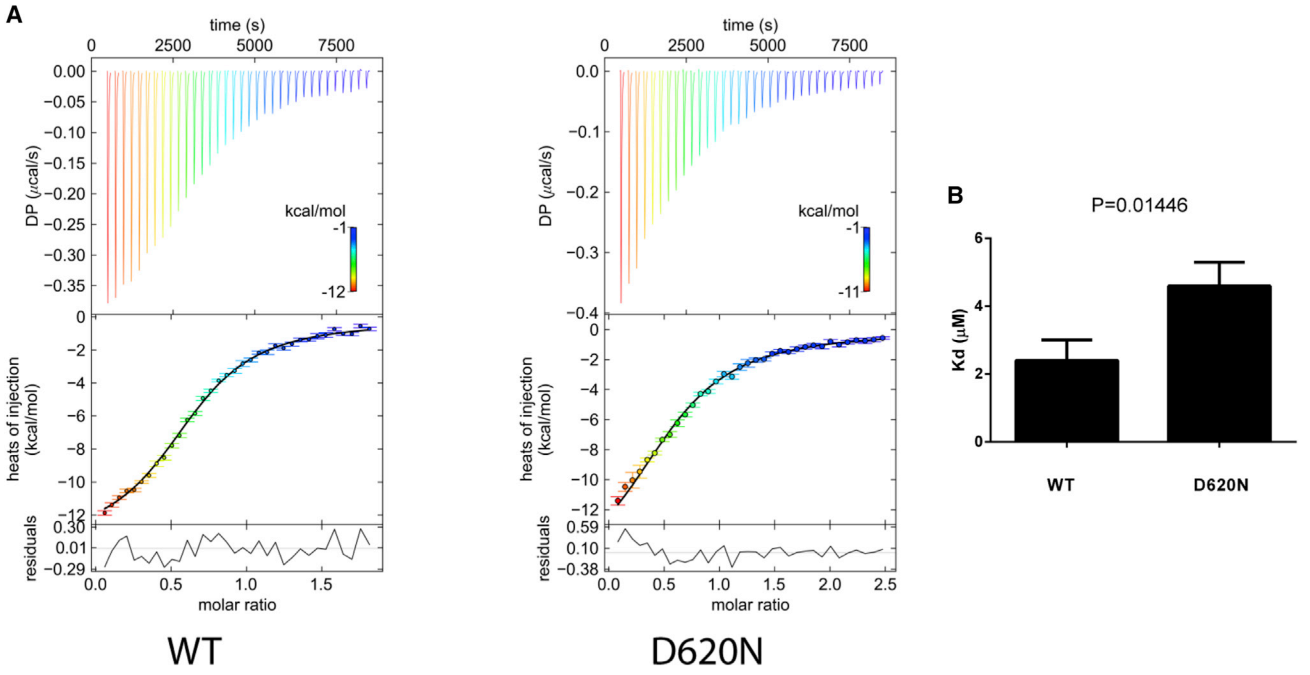

Figure 4. VPS35(D620N) Has Reduced Affinity for the FAM21 Tail

(A) Isothermal titration calorimetry of $FAM21_{R20-21}$ titrated into the VPS35/VPS29 complex. Top, middle, and bottom panels are from representative experiments showing raw, integrated heat, and fitting residuals, respectively. The black curve in the middle panel represents a fit of the integrated data. Left: $VPS35^{WT}$/VPS29. Right: $VPS35^{D620N}$/VPS29.

(B) Quantification of the dissociation constant (K_d) determined from three independent ITC experiments for each protein complex as shown in (A). All values are presented as mean ± SD, derived from software package SEDPHAT for a global fitting. Student's t test was used to determine the difference of the two sets of data.

RME-8, SDCCAG3, and/or ANKRD50 may also contribute to the defect in retromer function in Parkinson disease. As such, the role of these newly identified retromer interactors offers a potentially exciting avenue of research into the causes of this neurodegenerative disease.

Supplemental Information

Supplemental Information includes Supplemental Experimental Procedures, two figures, and two tables and can be found with this article online at http://dx.doi.org/10.1016/j.cub.2014.06.024.

Author Contributions

I.J.M, F.S., D.D.B., M.K.R., and P.J.C. designed the project; I.J.M. and F.S. performed the cell biology experiments; K.J.H. performed the proteomics; D.J. performed the biochemical analysis of the VPS35 FAM21 interaction; P.A.B., K.J.M., A.L.W., and M.A.C. generated the VPS35(D620N) patient fibroblast cell line. I.J.M., F.S., D.J., D.D.B., M.K.R., and P.J.C. wrote the manuscript.

Acknowledgments

We specifically thank Dr. Kai Erdmann (University of Sheffield) for supplying reagents to study SDCCAG3. I.J.M. was supported by Wellcome Trust 4 Year PhD Studentship awarded through the Dynamic Cell Biology programme (083474). P.J.C. is supported by the Wellcome Trust (089928 and 085743), the Medical Research Council (MR/K018299/1), and the Royal Society. D.D.B. is supported by NIH grant AI065474. M.K.R. and D.J. are supported by the Howard Hughes Medical Institute and grants from the NIH (R01-GM56322) and Welch Foundation (I–1544).

Received: November 24, 2013
Revised: May 16, 2014
Accepted: June 10, 2014
Published: June 26, 2014

References

1. Cullen, P.J., and Korswagen, H.C. (2012). Sorting nexins provide diversity for retromer-dependent trafficking events. Nat. Cell Biol. *14*, 29–37.
2. Vilariño-Güell, C., Wider, C., Ross, O.A., Dachsel, J.C., Kachergus, J.M., Lincoln, S.J., Soto-Ortolaza, A.I., Cobb, S.A., Wilhoite, G.J., Bacon, J.A., et al. (2011). VPS35 mutations in Parkinson disease. Am. J. Hum. Genet. *89*, 162–167.
3. Zimprich, A., Benet-Pagès, A., Struhal, W., Graf, E., Eck, S.H., Offman, M.N., Haubenberger, D., Spielberger, S., Schulte, E.C., Lichtner, P., et al. (2011). A mutation in VPS35, encoding a subunit of the retromer complex, causes late-onset Parkinson disease. Am. J. Hum. Genet. *89*, 168–175.
4. Arighi, C.N., Hartnell, L.M., Aguilar, R.C., Haft, C.R., and Bonifacino, J.S. (2004). Role of the mammalian retromer in sorting of the cation-independent mannose 6-phosphate receptor. J. Cell Biol. *165*, 123–133.
5. Carlton, J., Bujny, M., Peter, B.J., Oorschot, V.M., Rutherford, A., Mellor, H., Klumperman, J., McMahon, H.T., and Cullen, P.J. (2004). Sorting nexin-1 mediates tubular endosome-to-TGN transport through coincidence sensing of high- curvature membranes and 3-phosphoinositides. Curr. Biol. *14*, 1791–1800.
6. Seaman, M.N. (2004). Cargo-selective endosomal sorting for retrieval to the Golgi requires retromer. J. Cell Biol. *165*, 111–122.
7. Wassmer, T., Attar, N., Harterink, M., van Weering, J.R., Traer, C.J., Oakley, J., Goud, B., Stephens, D.J., Verkade, P., Korswagen, H.C., and Cullen, P.J. (2009). The retromer coat complex coordinates endosomal sorting and dynein-mediated transport, with carrier recognition by the trans-Golgi network. Dev. Cell *17*, 110–122.
8. Steinberg, F., Gallon, M., Winfield, M., Thomas, E.C., Bell, A.J., Heesom, K.J., Tavaré, J.M., and Cullen, P.J. (2013). A global analysis of SNX27-retromer assembly and cargo specificity reveals a function in glucose and metal ion transport. Nat. Cell Biol. *15*, 461–471.
9. Wieman, H.L., Horn, S.R., Jacobs, S.R., Altman, B.J., Kornbluth, S., and Rathmell, J.C. (2009). An essential role for the Glut1 PDZ-binding motif in growth factor regulation of Glut1 degradation and trafficking. Biochem. J. *418*, 345–367.

10. Follett, J., Norwood, S.J., Hamilton, N.A., Mohan, M., Kovtun, O., Tay, S., Zhe, Y., Wood, S.A., Mellick, G.D., Silburn, P.A., et al. (2014). The Vps35 D620N mutation linked to Parkinson's disease disrupts the cargo sorting function of retromer. Traffic *15*, 230–244.
11. MacLeod, D.A., Rhinn, H., Kuwahara, T., Zolin, A., Di Paolo, G., McCabe, B.D., Marder, K.S., Honig, L.S., Clark, L.N., Small, S.A., and Abeliovich, A. (2013). RAB7L1 interacts with LRRK2 to modify intraneuronal protein sorting and Parkinson's disease risk. Neuron *77*, 425–439.
12. Harbour, M.E., Breusegem, S.Y., Antrobus, R., Freeman, C., Reid, E., and Seaman, M.N. (2010). The cargo-selective retromer complex is a recruiting hub for protein complexes that regulate endosomal tubule dynamics. J. Cell Sci. *123*, 3703–3717.
13. Derivery, E., Sousa, C., Gautier, J.J., Lombard, B., Loew, D., and Gautreau, A. (2009). The Arp2/3 activator WASH controls the fission of endosomes through a large multiprotein complex. Dev. Cell *17*, 712–723.
14. Gomez, T.S., and Billadeau, D.D. (2009). A FAM21-containing WASH complex regulates retromer-dependent sorting. Dev. Cell *17*, 699–711.
15. Harbour, M.E., Breusegem, S.Y., and Seaman, M.N. (2012). Recruitment of the endosomal WASH complex is mediated by the extended 'tail' of Fam21 binding to the retromer protein Vps35. Biochem. J. *442*, 209–220.
16. Jia, D., Gomez, T.S., Billadeau, D.D., and Rosen, M.K. (2012). Multiple repeat elements within the FAM21 tail link the WASH actin regulatory complex to the retromer. Mol. Biol. Cell *23*, 2352–2361.
17. Hao, Y.H., Doyle, J.M., Ramanathan, S., Gomez, T.S., Jia, D., Xu, M., Chen, Z.J., Billadeau, D.D., Rosen, M.K., and Potts, P.R. (2013). Regulation of WASH-dependent actin polymerization and protein trafficking by ubiquitination. Cell *152*, 1051–1064.
18. Popoff, V., Mardones, G.A., Bai, S.-K., Chambon, V., Tenza, D., Burgos, P.V., Shi, A., Benaroch, P., Urbé, S., Lamaze, C., et al. (2009). Analysis of articulation between clathrin and retromer in retrograde sorting on early endosomes. Traffic *10*, 1868–1880.
19. Shi, A., Sun, L., Banerjee, R., Tobin, M., Zhang, Y., and Grant, B.D. (2009). Regulation of endosomal clathrin and retromer-mediated endosome to Golgi retrograde transport by the J-domain protein RME-8. EMBO J. *28*, 3290–3302.
20. Seaman, M.N., Harbour, M.E., Tattersall, D., Read, E., and Bright, N. (2009). Membrane recruitment of the cargo-selective retromer subcomplex is catalysed by the small GTPase Rab7 and inhibited by the Rab-GAP TBC1D5. J. Cell Sci. *122*, 2371–2382.
21. Girard, M., Poupon, V., Blondeau, F., and McPherson, P.S. (2005). The DnaJ-domain protein RME-8 functions in endosomal trafficking. J. Biol. Chem. *280*, 40135–40143.
22. Hagemann, N., Ackermann, N., Christmann, J., Brier, S., Yu, F., and Erdmann, K.S. (2013). The serologically defined colon cancer antigen-3 interacts with the protein tyrosine phosphatase PTPN13 and is involved in the regulation of cytokinesis. Oncogene *32*, 4602–4613.
23. Bi, F., Li, F., Huang, C., and Zhou, H. (2013). Pathogenic mutation in VPS35 impairs its protection against MPP(+) cytotoxicity. Int. J. Biol. Sci. *9*, 149–155.
24. Helfer, E., Harbour, M.E., Henriot, V., Lakisic, G., Sousa-Blin, C., Volceanov, L., Seaman, M.N., and Gautreau, A. (2013). Endosomal recruitment of the WASH complex: active sequences and mutations impairing interaction with the retromer. Biol. Cell *105*, 191–207.
25. Valdmanis, P.N., Meijer, I.A., Reynolds, A., Lei, A., MacLeod, P., Schlesinger, D., Zatz, M., Reid, E., Dion, P.A., Drapeau, P., and Rouleau, G.A. (2007). Mutations in the KIAA0196 gene at the SPG8 locus cause hereditary spastic paraplegia. Am. J. Hum. Genet. *80*, 152–161.
26. Vilariño-Güell, C., Rajput, A., Milnerwood, A.J., Shah, B., Szu-Tu, C., Trinh, J., Yu, I., Encarnacion, M., Munsie, L.N., Tapia, L., et al. (2014). DNAJC13 mutations in Parkinson disease. Hum. Mol. Genet. *23*, 1794–1801.
27. Zavodszky, E., Seaman, M.N.J., Moreau, K., Jimenez-Sanchez, M., Breusegem, S.Y., Harbour, M.E., and Rubinsztein, D.C. (2014). Mutation in VPS35 associated with Parkinson's disease impairs WASH complex association and inhibits autophagy. Nat Commun *5*, 3828.

Current Biology, Volume *24*

Supplemental Information

Retromer Binding to FAM21 and the WASH Complex Is Perturbed by the Parkinson Disease-Linked VPS35(D620N) Mutation

Ian J. McGough, Florian Steinberg, Da Jia, Peter A. Barbuti, Kirsty J. McMillan, Kate J. Heesom, Alan L. Whone, Maeve A. Caldwell, Daniel D. Billadeau, Michael K. Rosen, and Peter J. Cullen

Supplementary Figures

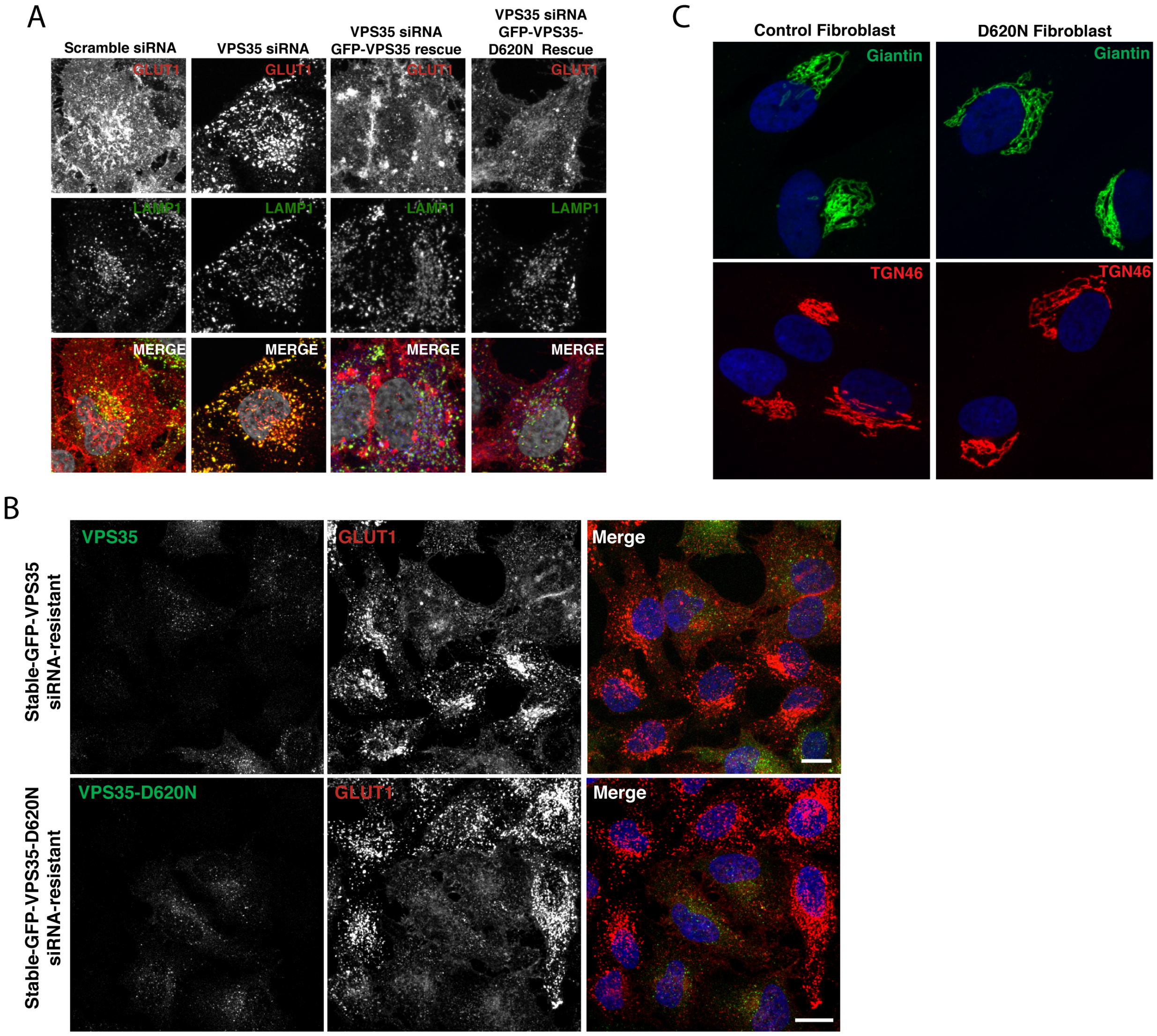

Supplementary Figure 1. The VPS35(D620N) mutation does not impair endosome-to-plasma membrane transport of GLUT1. A: GFP-VPS35(D620N) expression rescues the lysosomal accumulation of GLUT1 caused by VPS35 suppression. HeLa were transfected with non-targeting siRNA or VPS35 siRNA or transduced with lentiviruses encoding for RNAi resistant GFP-VPS35 or GFP-VPS35(D620N) and transfected with siRNA targeting endogenous VPS35. Cells were fixed and stained for VPS35 and GLUT1. Scale bar 10 μm. **B:** The experimental set-up is the same as in A but a larger field of view is shown for side by side comparison of rescue and non rescue cells. **C:** The Golgi is not fragmented in D620N fibroblast cells. The cell lines derived from healthy and D620N patients were fixed and stained for Giantin and TGN46.

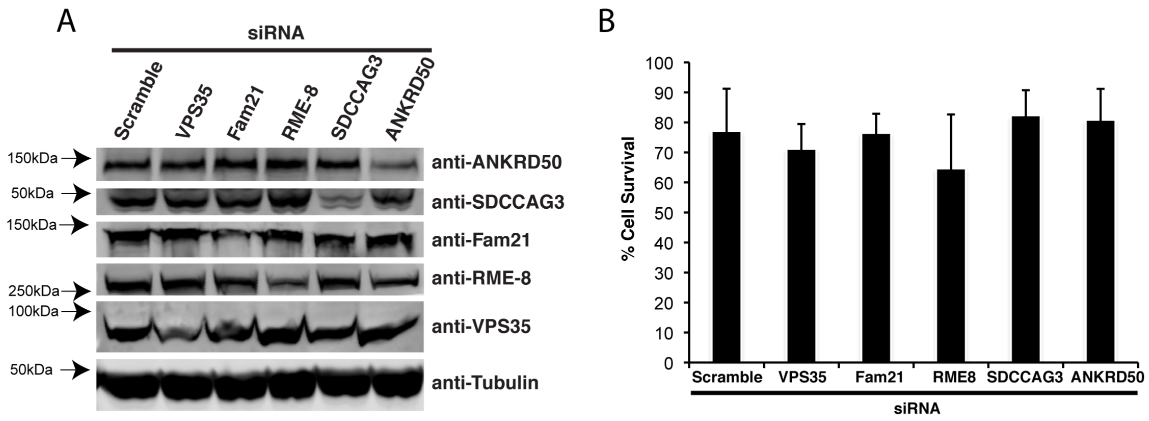

Supplementary Figure 2. Suppression of VPS35 or components identified as being decreased in the GFP-VPS35(D620N) immune-precipitations does not impair cell survival in the SH-SY5Y neuroblastoma cell line. A: SH-SY5Y cells were twice transfected with the indicated siRNAs, the second transfection 48 hours after the first. 24 hours after the second transfection 500 μM of MPP+ was added to the cells. 48 hours after addition of MPP+ cell viability was determined using a 3-(4, 5-dimethylthiazol-2-yl)-5-(3-carboxymethoxyphenyl)-2-(4-sulfophenyl)-2H-tetrazolium salt (MTS) assay according to manufacturer's instructions (Promega).

Supplementary Table 1. SILAC quantified interactome GFP-VPS35 vs. GFP-VPS35(D620N) from human RPE1 cells filtered to include only top heights (the cut-off for filtering was an enrichment of 5 fold or more and with 2 or more peptides quantified). Human RPE1 cells expressing either GFP, GFP-VPS35 or GFP-VPS35(D620N) were cultured in light DMEM (GFP), medium heavy (GFP-VPS35) or in heavy SILAC medium (GFP-VPS35(D620N)), followed by lysis and precipitation with GFP Nanotrap beads. After precipitation, beads were combined, bound proteins resolved by SDS-PAGE and subjected to detection and quantification of heavy and light peptides by LC-MS/MS.

Supplementary Table 2. Unfiltered SILAC quantified interactome GFP-VPS35 vs. GFP-VPS35(D620N) from human RPE1 cells.. The same data set as in Supplementary Table 1 but without the cut-off for enrichment and number of peptides detected. Human RPE1 cells expressing either GFP, GFP-VPS35 or GFP-VPS35(D620N) were cultured in light DMEM (GFP), medium heavy (GFP-VPS35) or in heavy SILAC medium (GFP-VPS35(D620N)), followed by lysis and precipitation with GFP Nanotrap beads. After precipitation, beads were combined, bound proteins resolved by SDS-PAGE and subjected to detection and quantification of heavy and light peptides by LC-MS/MS.

Supplementary Experimental Procedures

Antibodies.

Antibodies used in the study were: rabbit polyclonal WASH1 and FAM21 [15], rabbit polyclonal Strumpellin antibody (Santa Cruz, 87442), rabbit polyclonal VPS26 (Epitomics S1181) and VPS35 (Abcam 97545), mouse monoclonal GFP (clones 7.1/13.1, Roche 11814460001), rabbit polyclonal GLUT1 (Abcam 15309), rabbit monoclonal Itgβ1 (clone EO1042Y, Abcam, 52971), mouse monoclonal TfnR (clone H68.4, Invitrogen 13-6890), rabbit polyclonal LAMP1 (Abcam 24170), rabbit polyclonal RME-8 (gift from D. McPherson, McGill University, Montreal, Canada), rabbit polyclonal Varp (Abcam, ab108216), rabbit polyclonal ANKRD50 (Abcam, ab108219), mouse monoclonal CI-MPR (Abd Serotec, MCA2048T), sheep polyclonal TGN46 (Abd Serotec, AHP500), rabbit polyclonal SDCCAG3 (Proteintech 15969-1-AP), mouse monoclonal anti-β-actin (Applied Biological Materials) and goat anti-VPS35 antibodes (Abcam, 10099).

SILAC interactome analysis.

All SILAC reagents were sourced from Thermo Fisher; except dialysed FBS, which was from Sigma. Human RPE1 cells were grown in the SILAC DMEM for at least 6 passages to achieve full labelling. GFP–VPS35, GFP-VPS35(D620N) and GFP were lentiviral transduced before the labelling. Cells were lysed in precipitation buffer (50 mM Tris–HCl, 0.5% NP40, Roche Protease Inhibitor Cocktail) and GFP was precipitated with GFP-trap beads (Chromotek) for 30 min. Samples were separated on Nupage 4-12% precast gels (Invitrogen) and subjected to LC–MS/MS analysis on an Orbitrap Velos (Thermo) mass spectrometer as previously described [S1, S2].

Cell culture, transfection, immunofluorescence and western blot analysis

HeLa and RPE1 cells were maintained in DMEM (Gibco-Invitrogen) plus 10% (v/v) fetal calf serum (Sigma-Aldrich) and penicillin/streptomycin (PAA). Prior to MPP+ toxicity assays, cells were transfected twice, 48 hours apart, with the indicated siRNA. After the second transfection the cells were incubated in 500 μM MPP+ (Sigma) for 48 hours. Cell viability was determined using a 3-(4, 5-dimethylthiazol-2-yl)-5-(3-carboxymethoxyphenyl)-2-(4-sulfophenyl)-2H-tetrazolium salt (MTS) assay according to manufacturer's instruction (Promega). For immunofluorescence analysis, cells were fixed in 0.1 M phosphate buffer containing 4% (w/v) paraformaldehyde for 10 minutes on ice and permeabilized with 0.1% (v/v) Triton X-100 for 5 minutes. Thereafter, cells were incubated with 0.5% (w/v) BSA for 30 minutes followed by incubation with the indicated primary antibodies and subsequent incubation with secondary antibodies (Molecular Probes). For nuclear staining DAPI was used. Images were recorded on a Leica SPE or a Leica AOBS-SP2 confocal microscope. Following acquisition, images were analysed with the Volocity software package (Perkin Elmer): to filter noise, thresholds were applied uniformly across conditions. After setting of the thresholds, the percentage of co-localization and the Pearson's correlation between the respective channels were quantified with the co-localization tool of the Volocity software. Each co-localization analysis is based on the quantification of at least 150 images. For immuno-precipitation experiments the respective cell lines stably transduced with the desired GFP-tagged constructs were lysed in Tris-based immunoprecipitation buffer (50 mM Tris–HCl, 0.5% NP40 and Roche Protease inhibitor cocktail) and GFP precipitated with GFP-trap beads (Chromotek). Immuno-blottling was performed using standard procedures. Detection was carried out on a Licor Odyssey Infrared scanning system using fluorescently labelled secondary antibodies.

siRNA.

For VPS35 suppression the on-target plus human smartpool (Dharmacon) was used, except when suppressed in cells stably expressing a GFP-VPS35 siRNA resistant construct, in which case only oligo-3 from the smartpool was used. RME-8, ANKRD50 and FAM21 were suppressed using on-target plus human smartpools (Dharmacon). SDCCAG3 was suppressed using the following oligo: AAU UCU AAG CUG AGA AGA ATT.

Qantification of the surface levels of GLUT1

For the quantification of protein surface, cells were surface biotinylated with a commercially available kit (Pierce/Thermo) according to the manufacturer's instructions seventy-two hours post transfection. Cells were lysed in PBS with 2% Triton X-100, lysates were pooled and streptavidin agarose (GE-Healthcare) was used to capture biotinylated membrane proteins. After capture, biotinylated proteins were washed extensively in 1.2 M NaCl containing PBS with 1% Triton X-100 to remove cytoskeletal proteins and contaminants from the transmembrane proteins, followed by elution of the proteins by boiling in dithiothreitol-containing sample buffer.

Degradation assays.

To measure degradation of surface proteins, HeLa cells were transfected with the requisite siRNA. Twenty-four hours post transfection, before the effects of the siRNA were fully established, surface proteins were biotinylated with sulpho-NHS-biotin (Pierce/Thermo). Zero, twelve and twenty-four hours after biotinylation (24, 36 and 48 hours post transfection of siRNA), cells were lysed, biotinylated proteins were

captured with streptavidin agarose (GE-Healthcare) and detected by quantitative western blotting on an Odyssey scanner. Degradation was quantified as fluorescence signal remaining after 12 and 24 hours in percentage of signal intensity at time point 0.

VPS35 constructs and mutagenesis.

VPS35 was subcloned into pXLG3. shRNA viral construct against the 3'UTR of VPS35 was purchased from Sigma (TRCN0000337019). The D620N mutation (1858G>A) was generated using site-directed mutagenesis with the following primer 5'-GTA TGA AGA TGA AAT CAG CAA TTC CAA AGC-3'. siRNA resistant VPS35 was generated by introducing 6 silent base mis-matches (T1314A, T1317C, G1320A, C1321T, T1323A, T1326C) into the open reading frame, conferring resistance to siRNA VPS35-3 as described above.

Protein purification and isothermal titration calorimetry (ITC):

Protein purification and the ITC experiments were performed as previously described [S1, S2]. Briefly, equal amounts of BL21 (DE3) T1R cells (New England Biolabs, NEB) individually expressing GST-fusions of full-length VPS35 and VPS29 were mixed and co-lysed. The formed VPS35/VPS29 dimers were purified by glutathione-sepharose 4B affinity beads (GE Healthcare) and treated by TEV protease to remove the GST tag. The cleaved proteins were further purified by anion exchange (SOURCE 15Q, GE Healthcare) and size exclusion (Superdex 200, GE Healthcare) chromatographies. FAM21 fragments were expressed with a N-terminal TEV-cleavable MBP tag and a C-terminal, noncleavable His6 tag. The FAM21 proteins were purified by Amylose resin (NEB), subjected to TEV protease digestion, and by Ni-NTA resins (Qiagen). The purified VPS35/29 complex and FAM21 fragments were dialyzed in the same ITC buffer

(20 mM Tris, pH 8.0, 100 mM NaCl and 5 mM β-mercaptoethanol) for at least 24 hours at 4 °C. The ITC experiments were performed using a VP-ITC microcalorimeter (Microcal) at 20 °C by titrating 100 – 200 μM FAM21 proteins into 10 – 16 μM VPS35/VPS29 complex. Heat peaks were detected and integrated using NITPIC [S3]. The binding isotherms were globally fitted using SEDPHAT [S4]. Since the R20-21 fragment contains two binding sites for the VPS35/VPS29 complex, a 'two-site heterogeneous association (A+B+B=ABB)' model was chosen for fitting using the following variables: inclA (fraction of FAM21 incompetent for binding), log(Ka1) (logarithm of the macroscopic constant for the first binding), HAB (enthalpy change for the first binding). For each protein complex, the binding constants and thermodynamic parameters from three independent experiments were subjected to global fitting. We also tried to fit the ITC data with two different values for both Kd and enthalpy change, but could not obtain a statistically better fit. Finally, the limited amount of the data prevents us from analysing and reporting cooperativity.

Derivation of human VPS35(D620N) dermal fibroblasts from a skin biopsy:

Two 2 mm^2 skin biopsies were taken from the forearm of a healthy donor patient and a Parkinson's disease patient genotype harboring the VPS35(D620N) mutation. Each biopsy was placed into a sterile sample tube (Sterilin, UK) with approximately 10 ml of Fibroblast media. The samples were transported to the lab on wet ice in < 30 mins to be processed. In a class II hood, a sterile scalpel and forceps were used to remove the epidermis and any fat from the tissue and then cut into 4 pieces. These pieces are then placed into a 4 well plate (Nunc) pre-coated with CELLstart™ ((1:200 in PBS(+/+)), Sigma D8662) in 200 μl of Fibroblast media (DMEM (Sigma, D6546) supplemented with 10% Foetal Bovine Serum (Heat Inactivated), (Invitrogen, 10500), 1% Glutamax™ (Invitrogen, 35050), 1% NEAA (Invitrogen, 11140), 1% Penicillin-Streptomycin (Sigma,

P4458). The pieces were left to sit up to two weeks until they were attached to the matrix and the dermal fibroblasts started to proliferate. The cells were left to 80% confluency before being enzymatically passaged using TrypLE Express (Gibco, 12604). The cells were passaged at a ratio of 1:3 until cryopreserved.

Supplementary References

S1. Steinberg, F., Gallon, M., Winfield, M., Thomas, E., Bell, A.J., Heesom, K.J., Tavare, J.M., and Cullen, P.J. (2013). A global analysis of SNX27-retromer assembly and cargo specificity reveals a function in glucose and metal ion transport. Nat. Cell Biol. *15*, 461-471.

S2. Steinberg, F., Heesom, K.J., Bass, M.D., and Cullen, P.J. (2012). SNX17 protects integrins from degradation by sorting between lysosomal and recycling pathways. J. Cell Biol. *197*, 219-230.

S3. Keller, S., Vargas, C., Zhao, H., Piszczek, G., Brautigam, C.A., and Schuck, P. (2012). High-precision isothermal titration calorimetry with automated peak-shape analysis. Anal. Chem. *84*, 5066-5073.

S4. Houtman, J.C., Brown, P.H., Bowden, B., Yamaguchi, H., Appella, E., Samelson, L.E., and Schuck, P. (2007). Studying multisite binary and ternary protein interactions by global analysis of isothermal titration calorimetry data in SEDPHAT: application to adaptor protein complexes in cell signaling. Protein Sci. *16*, 30-42.
